# Disability-adjusted life years associated with population ageing in China, 1990-2017

**DOI:** 10.1186/s12877-021-02322-7

**Published:** 2021-06-16

**Authors:** Ruotong Li, Xunjie Cheng, David C. Schwebel, Yang Yang, Peishan Ning, Peixia Cheng, Guoqing Hu

**Affiliations:** 1 Department of Epidemiology and Health Statistics, Hunan Provincial Key Laboratory of Clinical Epidemiology, Xiangya School of Public Health, Central South University, Changsha, 410078 China; 2 Department of Geriatric Medicine, Xiangya Hospital, Central South University, Changsha, China; 3 Department of Psychology, University of Alabama at Birmingham, Birmingham, AL USA; 4 Department of Biostatistics, College of Public Health and Health Professions, University of Florida, Gainesville, FL USA; 5 Emerging Pathogens Institute, University of Florida, Gainesville, FL USA; 6 National Clinical Research Center for Geriatric Disorders, Xiangya Hospital, Central South University, Changsha, China

**Keywords:** Population ageing, Health burden, Disability adjusted life years, China

## Abstract

**Background:**

The Chinese population has aged significantly in the last few decades. Comprehensive health losses including both fatal and non-fatal health outcomes associated with ageing in China have not been detailed.

**Methods:**

Based on freely accessible disability adjusted life years (DALYs) estimated by the Global Burden of Diseases (GBD) 2017, we adopted a robust decomposition method that ascribes changes in DALYs in any given country across two time points to changes resulting from three sources: population size, age structure, and age-specific DALYs rate per 100,000 population. Using the method, we calculated DALYs associated with population ageing in China from 1990 to 2017 and examined the counteraction between the effects of DALYs rate change and population ageing. This method extends previous work through attributing the change in DALYs to the three sources.

**Results:**

Population ageing was associated with 92.8 million DALYs between 1990 and 2017 in China, of which 65.8% (61.1 million) were years of life lost (YLLs). Males had comparatively more DALYs associated with population ageing than females in the study period. The five leading causes of DALYs associated with population ageing between 1990 and 2017 were stroke (23.6 million), chronic obstructive pulmonary disease (COPD) (18.3 million), ischemic heart disease (13.0 million), tracheal, bronchus, and lung cancer (6.1 million) and liver cancer (5.0 million). Between 1990 and 2017, changes in DALYs associated with age-specific DALY rate reductions far exceeded those related to population ageing (− 196.2 million versus 92.8 million); 57.5% (− 112.8 million) of DALYs were caused by decreases in rates attributed to 84 modifiable risk factors.

**Conclusion:**

Population ageing was associated with growing health loss in China from 1990 to 2017. Despite the recent progress in alleviating health loss associated with population ageing, the government should encourage scientific research on effective and affordable prevention and control strategies and should consider investment in resources to implement strategies nationwide to address the future challenge of population ageing.

**Supplementary Information:**

The online version contains supplementary material available at 10.1186/s12877-021-02322-7.

## Introduction

As in many other countries, population ageing in China has emerged as an increasingly important social issue in the last few decades, driven especially by the combination of decreased birth rates and prolonged life expectancy [1]. A key step to address the societal public health challenge of ageing is to quantify the health impact associated with population ageing accurately [2].

Previous research has described changes in deaths, incidence, prevalence, and disability-adjusted life years (DALYs) associated with population ageing for specific diseases in particular countries and regions, including China [3–17]. However, these studies suffer from methodological limitations. Studies implementing traditional decomposition methods fail to generate robust results because those results are sensitive to the selections of decomposing order of population size, age structure and age-specific rate, as well as the choice of the reference group for comparisons [6, 7, 9, 10]. In addition, studies relying on projection models [4, 5, 8] depend heavily on the validity of model assumptions, which can deviate from reality and prevent researchers from properly distinguishing the effects of population ageing from that of population growth.

Another limitation of existing research is in the use of data from different sources and/or different time periods for decomposition analyses [15–17], making comparisons across studies difficult. In addition, many previous studies focus only on a single health outcome – either mortality [3, 5, 12], prevalence [9–11] or incidence [4, 6–8, 12] – each capturing only a slice of the health effects of population ageing.

Amidst the broader global literature, several studies from China offer evidence for the need to study population ageing among the Chinese population. A study by Xu et al. [11], for example, projected population ageing would cause significantly increased costs to treat dementia in China between 2020 and 2030. Three other studies quantified the impact of population ageing on cardiovascular incidences [12, 13] and deaths [14] in China. They reported an increase of coronary heart disease deaths related to population ageing in Beijing from 1990 to 2010 and predicted future cardiovascular incidence related to population ageing. One recent study quantified deaths related to population ageing in 195 countries/territories from 1990 to 2017, including China, and for 169 kinds of diseases and injuries [18]. While offering valuable data for the field, this study excluded the effect of population ageing on prevalence cases (non-fatal health outcomes) from the data analyses and did not cover decomposition details across sex and type of disease. Thus, assessing the impact of population ageing on a broad spectrum of health outcomes in China remains highly needed.

Developed from many different data sources and sophisticated statistical models, the Global Burden of Diseases, Injuries, and Risk Factors Study 2017 (GBD 2017) offers estimates of key health outcomes across 359 diseases and injuries, 84 modifiable risk factors or clusters of risks, and sex-specific population sizes for 195 countries and territories from 1990 to 2017 [19]. The dataset therefore provides comparable data across time periods and countries to permit accurate decomposition of the change in health outcomes associated population ageing.

Based on a recently-developed robust decomposition method [20], we employed estimates from the GBD 2017 to extend the literature by examining two research questions:

- Was the change in DALYs associated with population ageing in China from 1990 to 2017, and did the contribution of population ageing to the temporal change in DALYs vary by sex and by the type of disease?
- Did the changes in population ageing and change in the age-specific DALY rates, including overall rate and the attributed rate defined by GBD 2017, counteract each other in their effects on the overall DALY change from 1990 to 2017 in China, and did the level of counteraction vary by sex and by type of disease?

## Methods

### Data source

Data were derived from online resources of GBD 2017 [21]. The GBD study group integrates multiple-source data and adopts complex statistical models to mitigate the impact of limited availability and poor-quality of data (including underreporting, misclassification, and garbage codes) [19]. GBD estimates have been regularly updated since 2010 [22].

GBD 2017 estimates includes age- and sex-specific population sizes and key health indicators based on data from 1257 censuses and 761 population registry location-years [23, 24]. We used the level-3 categorization of causes defined by the GBD 2017, which includes 169 kinds of disease. Age was classified into 20 age groups from under-5 years to 95 years and older; each age group spanned 5 years.

We considered three primary outcomes. Years of life lost (YLLs) measure premature death and are calculated as the sum of the remaining life expectancy for people dying in each age group. Years lived with disability (YLDs) equal the sum of prevalent cases multiplied by the general public’s assessment of the severity of health loss. DALYs are calculated by summing YLDs and YLLs [19].

The research was approved by the Ethics Committee of Xiangya School of Public Health, Central South University, Changsha, China (approval number: XYGW-2020-50). Results are reported in adherence to the Guidelines for Accurate and Transparent Health Estimates Reporting (GATHER) statement (Addtional e-file-GATHER Checklist) [25].

### Decomposition method

We adopted a recently-developed decomposition method to analyze the data [20]. This method attributes differences for a given health outcome (e.g., death, DALY, YLD or YLL) between two time points to changes in three independent factors: (a) age structure of the population, the shifting of which toward greater numbers of older individuals are referred to as population ageing in the literature, (b) population size, and (c) age-specific rate, which can be interpreted as reflecting the joint effect of all factors other than age structure and population size. The decomposition process involves the following formulas:

1 $$ {M}_p={\sum}_{i=1}^{20}\left({N}_2-{N}_1\right){s}_{i1}{r}_{i1} $$

2 $$ {M}_a={\sum}_{i=1}^{20}{N}_1\left({s}_{i2}-{s}_{i1}\right){r}_{i1} $$

3 $$ {M}_r={\sum}_{i=1}^{20}{N}_1{s}_{i1}\left({r}_{i2}-{r}_{i1}\right) $$

4 $$ {I}_{pa}={\sum}_{i=1}^{20}\left({N}_2-{N}_1\right)\left({s}_{i2}-{s}_{i1}\right){r}_{i1} $$

5 $$ {I}_{pm}={\sum}_{i=1}^{20}\left({N}_2-{N}_1\right){s}_{i1}\left({r}_{i2}-{r}_{i1}\right) $$

6 $$ {I}_{am}={\sum}_{i=1}^{20}{N}_1\left({s}_{i2}-{s}_{i1}\right)\left({r}_{i2}-{r}_{i1}\right) $$

7 $$ {I}_{pam}={\sum}_{i=1}^{20}\left({N}_2-{N}_1\right)\left({s}_{i2}-{s}_{i1}\right)\left({r}_{i2}-{r}_{i1}\right) $$

Where *M*_*p*_, *M*_*a*_ and *M*_*r*_ represent the main effects of the changes in population size, age structure of the population and age-specific DALYs rates; *I*_*pa*_, *I*_*pr*_, *I*_*ar*_ and *I*_*par*_ indicate their one-way and two-way interactions; *s*_*ij*_ is the proportion of the *i*^th^ age group in population in the *j*^th^ year (*i* = 1, 2, ..., 20; *j* = 1, 2); *r*_*ij*_ denotes age-specific DALY rate of the *i*^th^ age group in the *j*^th^ year; and *N*_1_ and *N*_2_ represent the population size for the two population being compared. The change in the number of DALYs can then be attributed to changes in population ageing, population growth, and age-specific DALY rate as follows:

8 $$ A={M}_a+\raisebox{1ex}{$1$}\!\left/ \!\raisebox{-1ex}{$2$}\right.{I}_{am}+\raisebox{1ex}{$1$}\!\left/ \!\raisebox{-1ex}{$2$}\right.{I}_{pa}+\raisebox{1ex}{$1$}\!\left/ \!\raisebox{-1ex}{$3$}\right.{I}_{pa m} $$

9 $$ P={M}_p+\raisebox{1ex}{$1$}\!\left/ \!\raisebox{-1ex}{$2$}\right.{I}_{pm}+\raisebox{1ex}{$1$}\!\left/ \!\raisebox{-1ex}{$2$}\right.{I}_{pa}+\raisebox{1ex}{$1$}\!\left/ \!\raisebox{-1ex}{$3$}\right.{I}_{pa m} $$

10 $$ R={M}_r+\raisebox{1ex}{$1$}\!\left/ \!\raisebox{-1ex}{$2$}\right.{I}_{pm}+\raisebox{1ex}{$1$}\!\left/ \!\raisebox{-1ex}{$2$}\right.{I}_{am}+\raisebox{1ex}{$1$}\!\left/ \!\raisebox{-1ex}{$3$}\right.{I}_{pam} $$

A detailed introduction about the decomposition method is included in Additional e-file.

### Data analysis

We first graphed stacked bar charts to illustrate the associations of overall changes in DALYs, YLLs, and YLDs with population ageing from 1990 to 2017. Analogously, the associations were also plotted for the top ten level-3 disease categories defined by GBD 2017. Next, we compared changes in DALYs between 1990 and 2017 associated with changes in age-specific DALY rates and those changes associated with population ageing to assess possible counteraction between the effects of changes in DALY rates and population ageing. In addition, we performed the counteraction analysis for DALYs for each of the level-3 disease categories. Finally, we calculated DALYs attributable to the changes of 84 modifiable risk factors and those not attributable between 1990 and 2017 using the standard comparative risk assessment framework [24]. Similar analyses were conducted to assess the counteraction between changes in DALY rates attributed to 84 risk factors and population ageing.

Considering the substantial differences in aging and disease between males and females, we conducted analyses stratified by sex. We also conducted analyses separately for overall DALYs, YLLs, and YLDs. Data analyses were conducted using R version 3.6.1(R Core Team, 2020). Graphs were drawn using Microsoft Excel. R scripts for the decomposition method are included in the Additional e-file.

## Results

### Population ageing

GBD 2017 population estimates suggest the number of people aged 65 and older increased by 141.9% in China from 66 million (30 million males and 36 million females) in 1990 to 160 million (76 million males and 84 million females) in 2017 (Additional e-file). Over the same time period, the proportion of Chinese people aged 65 years and older rose from 5.5 to 11.3% (from 4.9 to 10.6% for males and from 6.2 to 12.1% for females) (Additional e-file).

### DALYs associated with population ageing

**Fig. 1:**
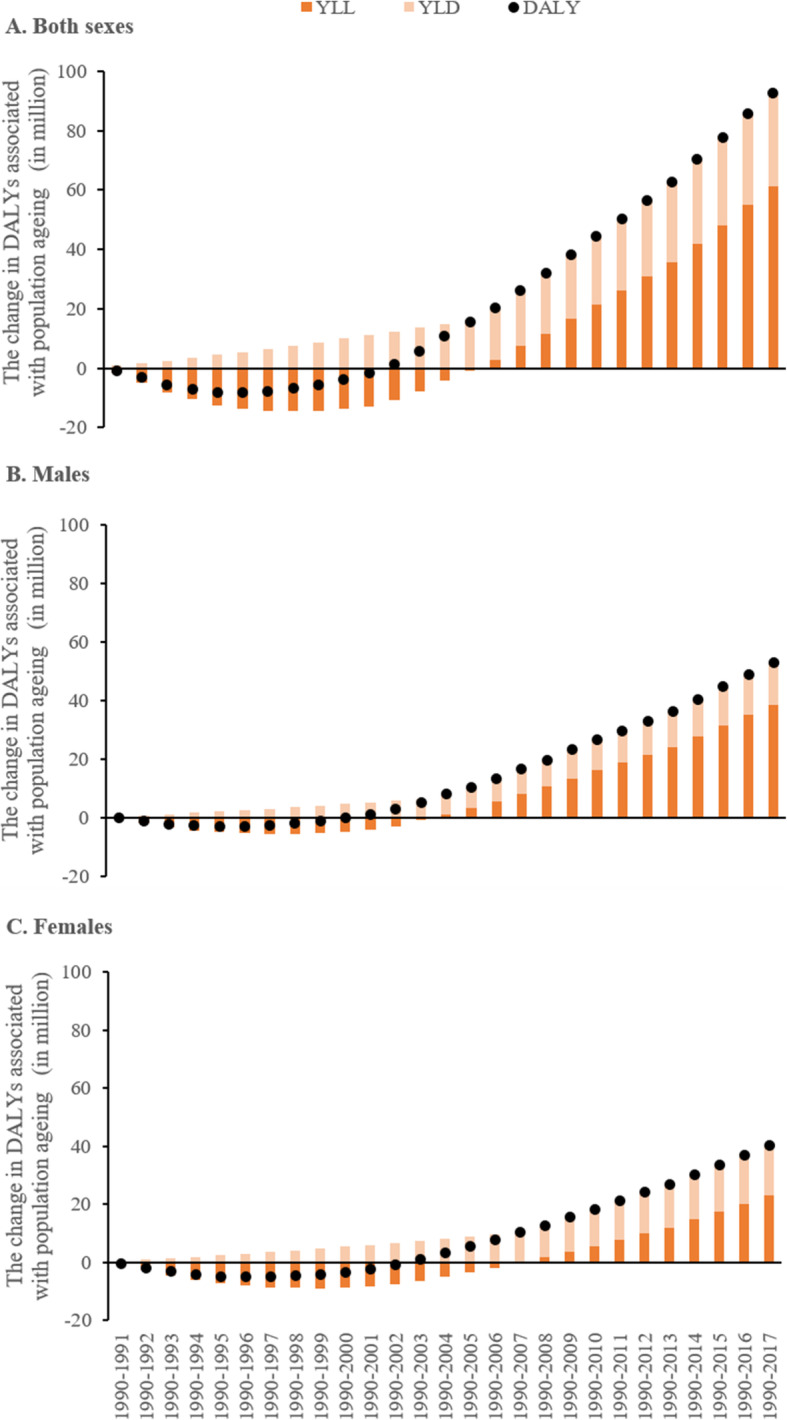
Change in disability-adjusted life years (DALYs) associated with population ageing in China, 1990-2017. Notes: YLLs - years of life lost, YLDs - years lived with disability, DALYs equals to the sum of YLLs and YLDs

Using year 1990 as the reference, DALYs associated with population ageing first decreased continuously from 1991 to 1996 and then began to increase gradually, reaching 92.8 million in 2017 (Fig. 1a). Notably, DALYs associated with population ageing remained less than zero from 1991 to 2001, as the result of similar changes in YLLs in this period. YLDs associated with population ageing gradually increased from 0.8 million to 31.7 million between 1991 and 2017 (using 1990 as reference), but its contribution to DALYs was exceeded by YLL after 2011. Between 1990 and 2017, YLLs accounted for 65.8% of DALYs (61.1 million) associated with population ageing, while YLDs explained the remaining 34.2% (31.7 million).

Males and females demonstrated similar patterns of DALY changes associated with population ageing from 1991 to 2017, using 1990 as the reference (Figs. 1b and 1c). Since 2000, however, the number of DALYs associated with population ageing was higher among males than among females. The contribution of YLDs to DALYs was higher among females than among males throughout the most recent decade, and reached 42.9% for females vs. 27.1% for males in 2017.

**Fig. 2:**
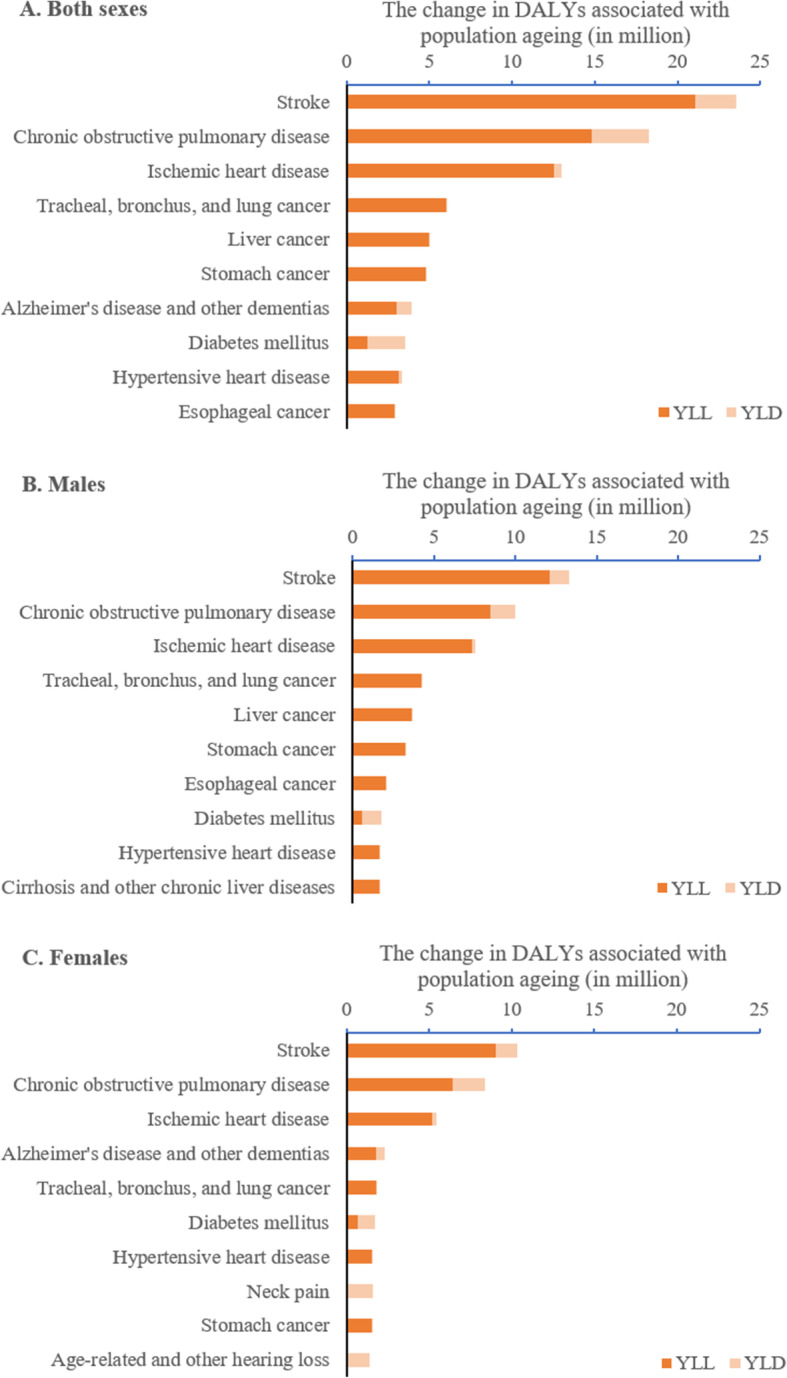
Top 10 diseases with the largest disability-adjusted life years (DALYs) change associated with population ageing between 1990 and 2017 in China. Notes: YLLs - years of life lost, YLDs - years lived with disability, DALYs equals to the sum of YLLs and YLDs

Changes in DALYs associated with population ageing between 1990 and 2017 varied across the 169 level-3 disease categories. The top ten categories accounted for 66.0 million (71.1%) of DALYs associated with population ageing. The five leading causes were stroke (23.6 million), chronic obstructive pulmonary disease (COPD) (18.3 million), ischemic heart disease (13.0 million), tracheal, bronchus, and lung cancer (6.1 million) and liver cancer (5.0 million) (Fig. 2a). YLLs explained more than 90% of DALYs associated with population ageing for the top ten causes except for stroke (89.4%), COPD (81.1%), Alzheimer’s disease and other dementia (77.3%), and diabetes mellitus (36.5%).

Although the spectrum of the top ten disease categories of changes in DALYs associated with population ageing between 1990 and 2017 was generally similar between males and females (Fig. 2b and 2c), the ordering of the top ten categories was somewhat different across the two sexes. For example, Alzheimer’s disease was ranked the 4th leading cause of DALYs for females (explaining 2.3 million DALYs) but the 11th leading cause for males (explaining 1.6 million DALYs). For the same diseases, males typically had more DALYs associated with population ageing compared to females.

### DALYs associated with rate changes versus with population ageing

**Fig. 3:**
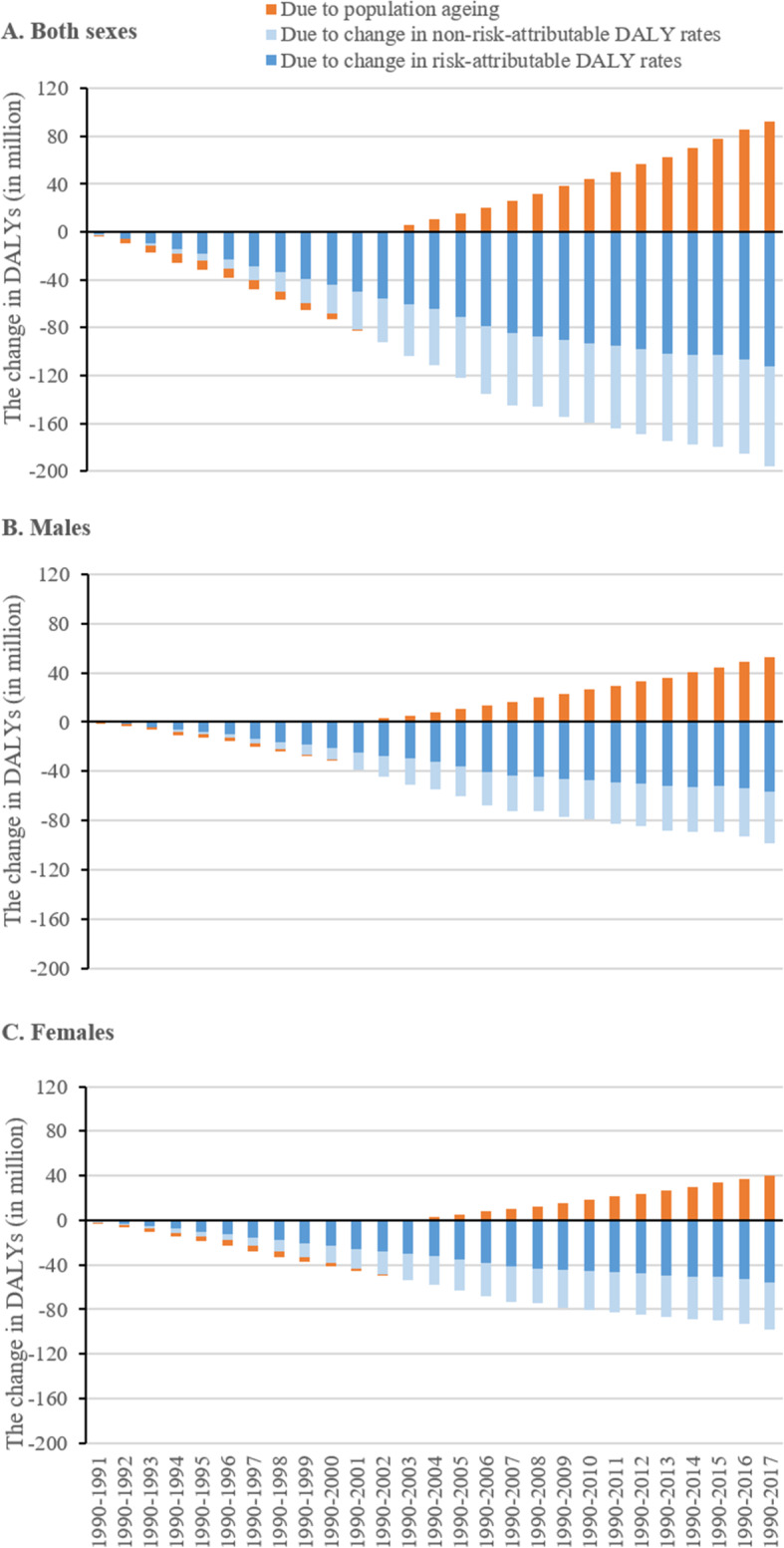
Change in disability-adjusted life years (DALYs) associated with rate changes versus population ageing in China, 1990-2017. Notes: DALYs - disability-adjusted life years

Between 1990 and 2017, DALYs associated with reductions in age-specific DALY rates far exceeded those related to population ageing (− 196.2 versus 92.8 million) (Fig. 3a). The overall temporal pattern of DALYs is jointly shaped by the counteracting effects of population ageing and reductions in age-specific DALY rates (Additional e-file Fig. S4). We further decomposed age-specific DALY rates into the rates that can be attributed to the 84 modifiable risk factors and the rates that cannot, allowing us to calculate changes in DALYs associated with changes in risk-attributable rates and in non-risk-attributable rates. Changes in DALYs driven by these two types of DALY rates are shown as dark and light blue stacked bars in Fig. 3. Changes in overall and risk-attributable DALY rates contributed more to total DALY changes than population ageing for nearly all years in the study period. Of the reduction of 196.2 million DALYs between 1990 and 2017 caused by rate reduction, 57.5% (− 112.8 million) was contributed by falling risk-attributable rates. Sex-specific analyses produced similar results (Fig. 3b and 3c).

**Fig. 4:**
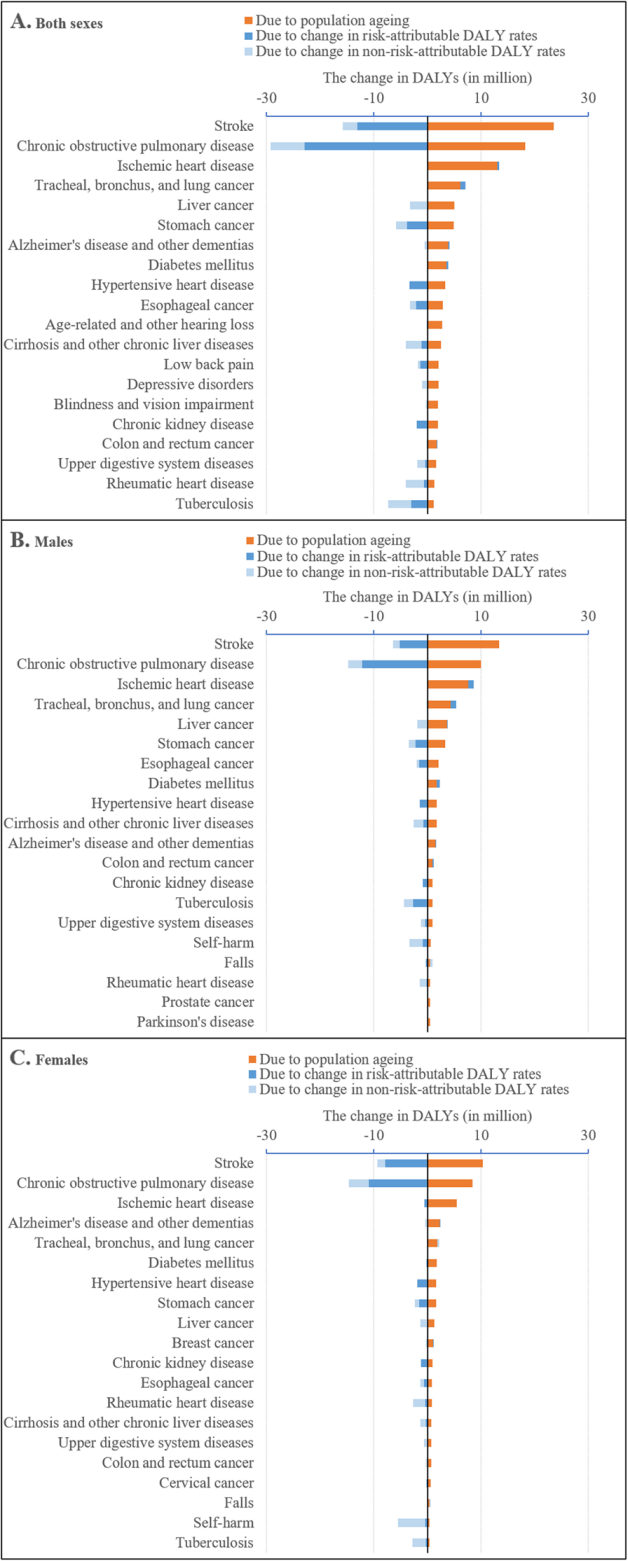
Disability-adjusted life years (DALYs) change associated with population ageing, change in risk-attributable DALY rates and change in non-risk-attributable DALY rates by sex among top 20 diseases most affected by population ageing in China, 1990–2017. Notes: 1. DALY change associated with change in risk-attributable DALY rates in this study refers to decrease of age-specific rate that can be explained by attributable GBD risk factors. 2. Diseases included in this part must be attributable and the number of DALY change associated with population ageing are greater than zero

The counteracting effect between reduced age-specific DALY rates and population ageing varied widely across level-3 disease categories (Fig. 4a). Among the top 20 disease categories with the largest changes in DALYs between 1990 and 2017 associated with population ageing for both sexes, the counteraction was prominent for nine categories: COPD (− 29.3 vs. 18.3 million), stomach cancer (− 5.8 vs. 4.9 million), hypertensive heart disease (− 3.4 vs. 3.3 million), esophageal cancer (− 3.3 vs. 2.9 million), cirrhosis and other chronic liver diseases (− 4.0 vs. 2.5 million), chronic kidney disease (− 2.0 vs. 1.9 million), upper digestive system diseases (− 1.9 vs. 1.6 million), rheumatic heart disease (− 4.0 vs. 1.3 million) and tuberculosis (− 7.3 vs. 1.2 million). That pattern did not hold for the other types of disease associated with the largest DALYs related to population ageing, including stroke (− 15.8 vs. 23.6 million) and ischemic heart disease (0.3 vs. 13.0 million). Further decomposition analyses showed that the effect of population ageing was surpassed by that of the falling risk-attributable DALY rates for four disease categories: COPD (− 23.0 vs. 18.3 million), hypertensive heart disease (− 3.4 vs. 3.3 million), chronic kidney disease (− 2.0 vs. 1.9 million) and tuberculosis (− 3.0 vs. 1.2 million).

When data were analyzed separately for males and females, we observed counteracting effects between falling DALY rates and population ageing for seven disease categories among males and for nine disease categories among females (Fig. 4b and c). Surprisingly, on occasion the magnitude of counteraction differed between males and females even for the same disease. For example, the contributions of falling DALY rates and population ageing for rheumatic heart disease were − 1.4 vs. 0.5 million for males and − 2.7 vs. 0.8 million for females.

## Discussion

This study presents four major findings that are novel to the field. First, an increase of 92.8 million DALYs was associated with population ageing in China between 1990 and 2017, with 65.8% of that increase in YLLs. Second, in China, males suffered more health losses associated with population ageing compared to females between 1990 and 2017 (52.8 vs. 40.4 million); stroke, chronic obstructive pulmonary disease and ischemic heart disease were most affected by population ageing, contributing 54.9 million (59.2%) to total DALY loss. Third, the increase in DALYs related to population ageing between 1990 and 2017 was offset by the decrease in DALYs due to reduced DALY rates (including overall rate and -risk-attributable rates). Last, the counteracting effect between decreasing DALY rates and population ageing was roughly similar between males and females but varied greatly across the level-3 disease categories.

Extending previous research on decomposing mortality changes in China [18], this study offers an overall picture of the health impact of population ageing in China using DALY as a metric. DALY offers a summary measure of morbidity, disability, and mortality, and therefore reflects overall health impact of diseases and injuries in a country compared to single fatal or non-fatal health indicators. The decomposition results obtained via applying a robust method [20] to GBD 2017 data is a valuable addition to the literature [12–14], providing rigorous estimates for China that can readily be compared across different time periods and with other countries for a variety of health outcomes [19]. We recommend the same decomposition method for analyzing GBD 2017 estimates for other countries to maximize comparability.

The small fluctuation in DALYs associated with population ageing we discovered before 2002 might be primarily due to the joint effects of temporal variations of both age structure and DALY rates across age groups in the population. For example, the proportion of people aged 30 to 65 with a fairly low DALY rate increased from 35.6 to 45.9% between 1990 and 2002, and the proportion of the under-five age group with an extremely high DALY rate decreased from 13.4 to 6.6% in the same time period.

The steady increase of DALYs associated with population ageing since 2002 was primarily caused by extremely high DALY rates among older people and the rise of the number of older people in the population rather than changes in population size or changes in age-specific rates. Ageing typically is related to progressive loss of physiological integrity, leading to impaired functions and increased vulnerability to morbidity and mortality among people in the oldest age groups [26, 27]. The increase in numbers of older people in China corresponds with prolonged life expectancy, which is attributed to substantial social development, especially improvement in health care services [28]. Between 1990 and 2017, the life expectancy of Chinese people rose from 68.7 to 70.1 years [29].

Interestingly, DALYs associated with population ageing in China were significantly larger among males than among females even though females had a comparatively higher life expectancy, a higher proportion of old people, and a larger number of old people. This finding reflects the comparatively higher DALY rate among males (29,019 per 100,000 population in 2017) compared to females (23,464 per 100,000 population) [21]. Similarly, variations in health losses associated with population ageing across different disease categories reflect varying changes in DALY rates.

Encouragingly, the health losses associated with population ageing in China were wholly counteracted by the reduction in DALY rates over time. Notably, the reduction of risk-attributable rates adequately offset the increase in DALYs associated with population ageing for COPD, hypertensive heart disease, chronic kidney disease and tuberculosis. This reflects considerable progress in prevention and control efforts for certain diseases such as COPD and stomach cancer. Progress has been inconsistent across disease categories, however, diseases like ischemic heart disease and diabetes should be targeted for successful ageing because the earn from reductions in DALY rates was insufficient to offset the health loss from population aging, and continued work is needed to control preventable health outcomes through evidenced-based strategies like prohibiting smoking in public places, reducing harmful use of alcohol, and promoting physical activity that have potential to continue such progress [30–32].

For diseases with substantial burden that is difficult to control, like dementia and age-related hearing loss, DALY rate reductions between 1990 to 2017 were minimal, and insufficient to offset the burden associated with population ageing. Continued research to understand and ultimately prevent such diseases is recommended [33, 34].

Our findings have three major implications. First, they demonstrate the enormity of health loss associated with population ageing in China. As life expectancy continues to rise, we might expect increased health losses in the future. This calls for urgent action to address the challenges of an ageing society. Second, our results illustrate how the health burden associated with population ageing can be offset by prevention and treatment efforts. Holistic efforts should be made to reduce population exposures to known modifiable risk factors for diseases. These efforts will require government investments to disseminate effective strategies that are available but not fully implemented nationwide; examples include creating age-friendly products and a built environment and promoting a healthy lifestyle among older adults [35]. Last, our findings underscore the value of increasing government investment in scientific research. Research is needed to generate effective prevention and treatment strategies, including for currently unpreventable diseases, and to explain observed changes in DALY rates that cannot be attributed to the known modifiable risk factors.

The primary limitation of this study is in its use of the GBD 2017 dataset. As previously noted [36–38], GBD 2017 estimates are affected by the paucity and low quality of raw data although advanced and complex models have been adopted to impute missing values and optimize the estimation for each country or territory, including China. Furthermore, because full posterior samples of cause-specific rates stratified by age sex, location, and year are not freely accessible for GBD 2017 [19], we were unable to provide 95% uncertainty intervals for the estimates.

## Conclusion

The increase in population ageing in China since the early 2000s was associated with substantial and growing DALY burden. Health losses associated with population ageing in China between 1990 and 2017 differed by sex and type of disease. The health burden associated with population ageing was largely offset by decreases in DALY rates associated with evidence-based prevention and treatment of diseases. The government of China – as well as the governments of other countries facing similar demographic patterns – should increase support for research on disease prevention and control and implement evidence-based intervention strategies to address the rising health losses associated with population ageing. For both the overall DALY and many disease-specific DALYs, a significant part of reduced DALY was not attributed to known modifiable risk factors for the overall rate as well as for many disease-specific rates, meriting further research to exploring the underlying drivers and to develop practical and generalizable interventions.

## Supplementary Information

**Additional file 1.**

## Data Availability

The data that support the findings of this study are available from Global Burden of Diseases, Injuries, and Risk Factors Study 2017, which is an open access resource. Bona fide researchers can apply to use the GBD 2017 data at GBD Results Tool: http://ghdx.healthdata.org/gbd-results-tool.
