# Supplementary material for: Disability-adjusted life years associated with population ageing in China, 1990-2017
Source: BMC Geriatr. 2021 Jun 16;21:369. doi: 10.1186/s12877-021-02322-7 (PMC8207592; doi:10.1186/s12877-021-02322-7)
Supplement: Supplementary file 1 — Additional file 1. [file 12877_2021_2322_MOESM1_ESM.docx]

**Supplementary Information for**

Disability-adjusted life years associated with population ageing in China, 1990-2017.

**This file includes:**

Supplementary text (including details of decomposition method)

R script for the decomposition method

Figures S1 to S4

Tables S1 to S6

GATHER Checklist

Supplementary References

Supplementary Information Text

1. **The decomposition method.**

The decomposition method used in this study was reported to be robust to the choice of decomposition order of the three factors (population size, age structure/population ageing, age-specific rate) and the choice of reference group, compared to the two most commonly used alternative methods^1^. This method was previously applied to quantify the impact of population ageing on mortality for 195 countries/territories and 169 causes of death^2^.

We demonstrate the calculation of disability-adjusted life years (DALYs) attributed to the three factors below. Age is divided using 5-year increments, from under-5 years old to 95 years and older. Let *d_ij_*, *n_i_*_j_, *r_ij_* and *s_ij_* denote the total number of DALYs, population size, age-specific DALY rate, and proportion of population for the *ith* age group of the *jth* year, respectively, (*i* = 1, 2, …, 20; *j* = 1, 2). Let *D_1_* and *D_2_*, *N_1_* and *N_2_*, *R_1_* and *R_2_* represent the total number of DALYs, population size and DALY rate for years 1990 and 2017 based on the following formulas:

$D_{1}=\sum_{i=1}^{20} d_{i1}$ (1)

$D_{2}=\sum_{i=1}^{20} d_{i2}$ (2)

$N_{1}=\sum_{i=1}^{20} n_{i1}$ (3)

$N_{2}=\sum_{i=1}^{20} n_{i2}$ (4)

$R_{1}={D_{1}}/{N_{1}}$ (5)

$R_{2}={D_{2}}/{N_{2}}$ (6)

$r_{ij}={d_{ij}}/{n_{ij}}$ (7)

$s_{ij}={n_{ij}}/{N_{j}}$ (8)

We use *M_p_*, *M_a_* and *M_r_* to represent the main effects of the changes in population size, in age structure and in DALYs rates, and *I_pa_*, *I_pr_*, *I_ar_* and *I_par_* to represent their one-way and two-way interactions, respectively. These terms are calculated as follows when using year 1990 as the reference:

$M_{p}=\sum_{i=1}^{20} {{\left( N_{2}-N_{1} \right)s}_{i1}r}_{i1}$ (9)

$M_{a}=\sum_{i=1}^{20} N_{1}\left( s_{i2}-s_{i1} \right)r_{i1}$ (10)

$M_{r}=\sum_{i=1}^{20} {N_{1}s}_{i1}\left( r_{i2}-r_{i1} \right)$ (11)

$I_{pa}=\sum_{i=1}^{20} \left( N_{2}-N_{1} \right)\left( s_{i2}-s_{i1} \right)r_{i1}$ (12)

$I_{pm}=\sum_{i=1}^{20} \left( N_{2}-N_{1} \right)s_{i1}\left( r_{i2}-r_{i1} \right)$ (13)

$I_{am}=\sum_{i=1}^{20} N_{1}\left( s_{i2}-s_{i1} \right)\left( r_{i2}-r_{i1} \right)$ (14)

$I_{pam}=\sum_{i=1}^{20} \left( N_{2}-N_{1} \right)\left( s_{i2}-s_{i1} \right)\left( r_{i2}-r_{i1} \right)$ (15)

The change in the number of DALYs can then be attributed to changes in population ageing, population growth, and age-specific DALY rate as follows:

$A{=M}_{a}+½I_{am}+½I_{pa}+⅓I_{pam}$ (16)

$P{=M}_{p}+½I_{pm}+½I_{pa}+⅓I_{pam}$ (17)

$R{=M}_{r}+½I_{pm}+½I_{am}+⅓I_{pam}$ (18)

2. Number and proportion of people aged 65 years and older

According to GBD 2017 population estimates, the number of people aged 65 and older in China increased by 141.9%, from 66 million (30 million males and 36 million females) in 1990 to 160 million (76 million males and 84 million females) in 2017 (**Fig.S1A**). Consequently, the proportion of Chinese people aged 65 years and older rose from 5.5% to 11.3% between 1990 and 2017 (from 4.9% to 10.6% for males and from 6.2% to 12.1% for females) (**Fig.S1B**).

**3. DALYs and age-specific DALY rate**

DALYs from all diseases in China was about 410 million (221 million in males, 189 million in females) in 1990. Over the next 27 years, the number of DALYs first decreased to 336 million in 2007 (189 million in males, 147 million in females), and then began to rise steadily, reaching 373 million (210 million in males, 163 million in females) in 2016 and flattening in 2017 (**Fig.S2**). Between 1990 and 2017, years of life lost (YLLs) consistently constituted the majority of DALYs (74.0%- 59.0%), and DALYs in males were more than in females across all years.

DALY rates decreased significantly between 1990 and 2017 for both sexes and all age groups, with the largest reduction in under-5 children (from 92,780 to 19,355 per 100,000 population) (**Fig.S3**).

**4. DALYs associated with population ageing, population growth and change in age-specific DALY rates**

Using 1990 as the baseline, DALYs associated with population ageing first decreased continuously from 1991 to 1996 and then began to increase gradually, reaching 92.8 million in 2017. Between 1990 and 2017, population growth was associated with an increase of 64.9 million DALYs, while age-specific DALY rates change was associated with a decrease of 196.2 million DALYs (**Fig.S4**).

**R script for the decomposition method**

setwd("C:/Users/liruo/Desktop/DALY total")

diseasesnames<-as.character(read.csv("diseasenames.csv",header = F)[,1])

calyear<-as.character(c(9091,9092,9093,9094,9095,9096,9097,9098,9099,

9000,9001,9002,9003,9004,9005,9006,9007,9008,

9009,9010,9011,9012,9013,9014,9015,9016,9017))

calresult<-c("pop","as","asmr")

nameresult<-paste(rep(calresult,each=27),rep(calyear,3),sep = "")

setwd("C:/Users/liruo/Desktop/population/result")

countrypop<-read.csv("Both sex pop.csv",header = T)

setwd("C:/Users/liruo/Desktop/DALY total")

countrydisease<-read.csv("trans.csv",header = T)

attributeresult<-matrix(0,nrow=170,ncol = 81)

for(icalculate in 1:170){

for(jcalculate in 1:27){

calculatematrix<-matrix(c(countrypop[,1],

countrypop[,(jcalculate+1)],

countrydisease[((20*icalculate-19):(20*icalculate)),2],

countrydisease[((20*icalculate-19):(20*icalculate)),(jcalculate+2)])

,nrow = 20,ncol = 4)

calculatematrix<-matrix(as.numeric(calculatematrix),nrow = 20,ncol = 4)

p1<-sum(calculatematrix[,1])

p2<-sum(calculatematrix[,2])

n1<-sum(calculatematrix[,3])

n2<-sum(calculatematrix[,4])

s1<-calculatematrix[,1]/p1

s2<-calculatematrix[,2]/p2

r1<-calculatematrix[,3]/calculatematrix[,1]

r2<-calculatematrix[,4]/calculatematrix[,2]

s<-p1*(s2-s1)*r1

r<-p1*(r2-r1)*s1

p<-(p2-p1)*r1*s1

sr<-(s2-s1)*(r2-r1)*p1

sp<-(s2-s1)*(r1)*(p2-p1)

pr<-(s1)*(r2-r1)*(p2-p1)

spr<-(s2-s1)*(r2-r1)*(p2-p1)

pp1<-sum(p+1/2*sp+1/2*pr+1/3*spr)

ss1<-sum(s+1/2*sp+1/2*sr+1/3*spr)

rr1<-sum(r+1/2*pr+1/2*sr+1/3*spr)

attributeresult[icalculate,jcalculate]<-round(pp1,2)

attributeresult[icalculate,(jcalculate+27)]<-round(ss1,2)

attributeresult[icalculate,(jcalculate+54)]<-round(rr1,2)

}

}

setwd("C:/Users/liruo/Desktop/DALY total")

rownames(attributeresult)<-diseasesnames

colnames(attributeresult)<-nameresult

write.csv(attributeresult,"both sex absolute.csv")


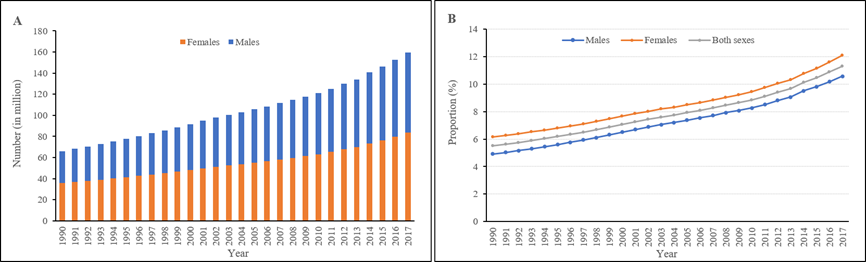


Fig. S1. Number and proportion of people aged 65 years and older in China, 1990-2017.


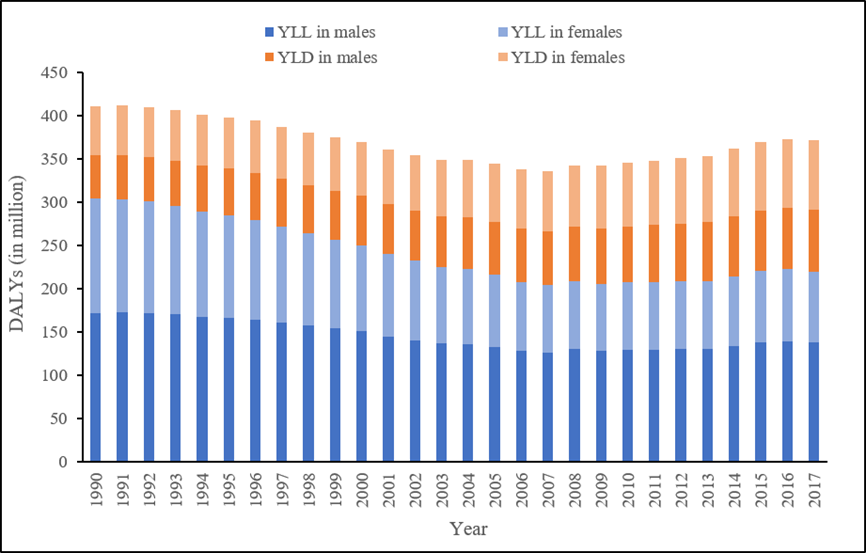


Fig. S2. Disability-adjusted life years (DALYs) in China, 1990-2017. YLL: years of life lost; YLD: Years lived with disability; DALYs equals to the sum of YLL and YLD.


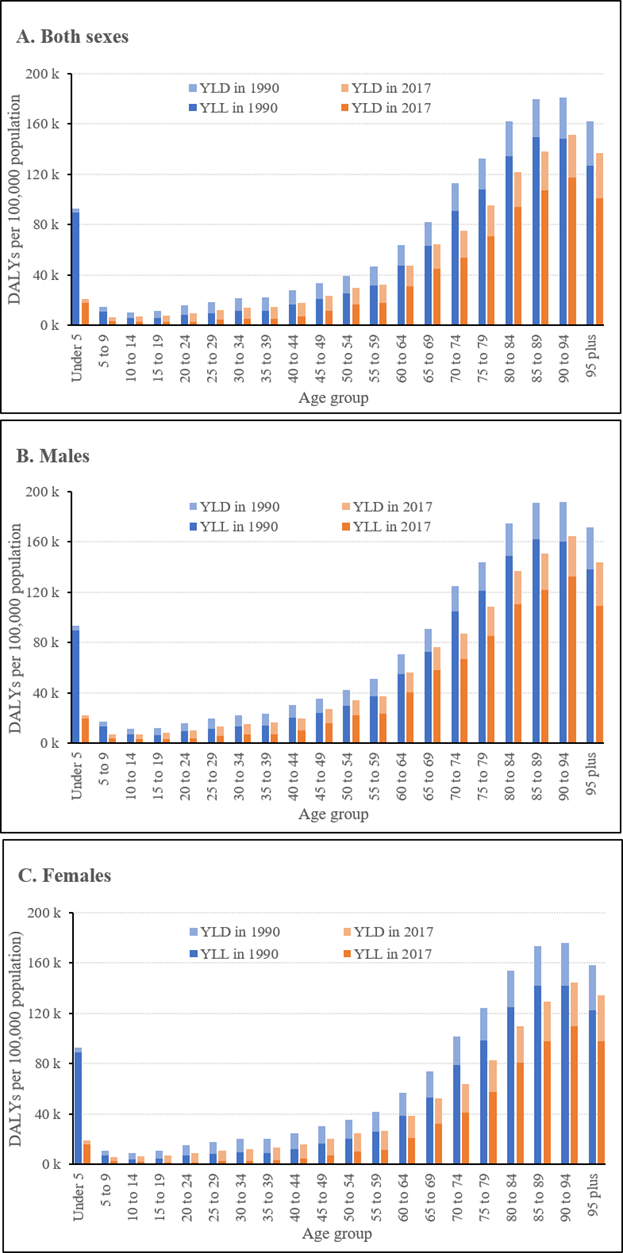


Fig. S3. Age-specific disability-adjusted life years (DALYs) rate by sex and age group in 1990 and 2017 for China. YLL: years of life lost; YLD: Years lived with disability; DALYs equals to the sum of YLL and YLD.


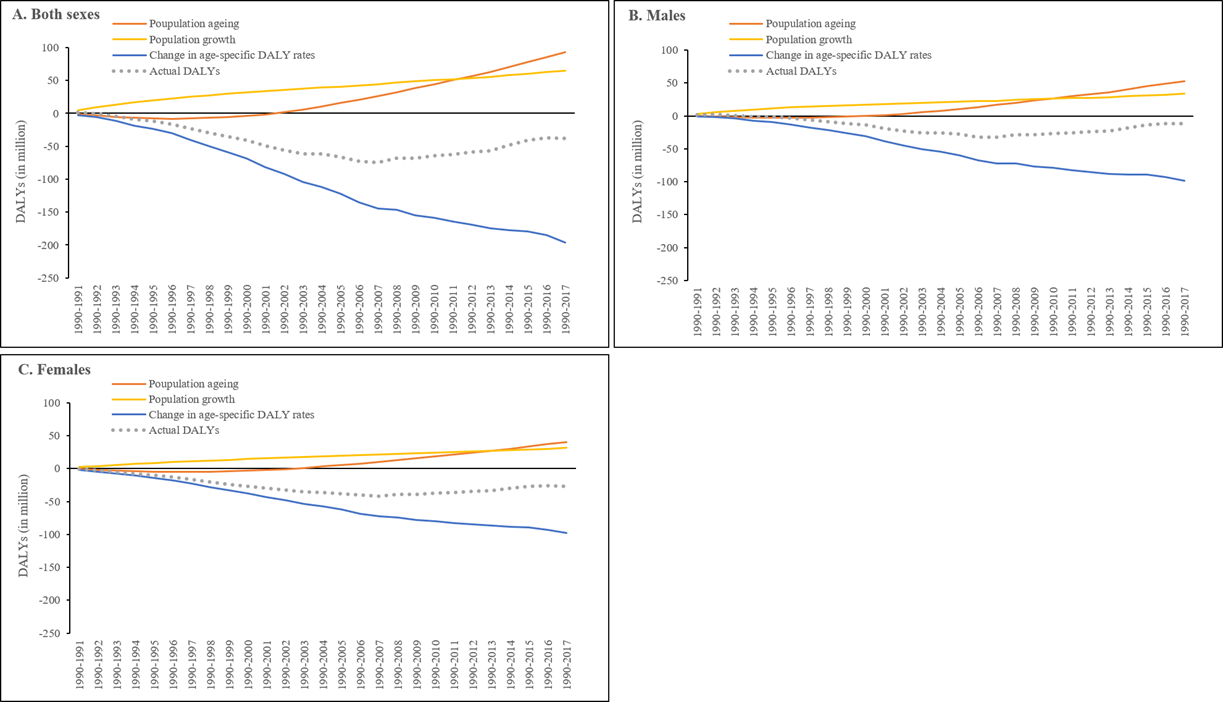


Fig. S4. DALYs associated with population, population growth, and change in DALY rate alternation from 1990 to 2017 in China. The decomposition was conducted using the number of DALYs in 1990 as the reference for each year.

Table S1. Total DALYs and DALYs, YLLs, YLDs associated with population aging between 1990 and 2017 by type of disease for both sexes in China.

| Type of disease | Change in DALYs  （in million） | Estimates associated with population ageing | | |
| --- | --- | --- | --- | --- |
|  |  | DALYs  (in million） | YLLs  (in million） | YLDs  (in million） |
| Stroke | 14.12 | 23.58 | 21.07 | 2.50 |
| Chronic obstructive pulmonary disease | -6.52 | 18.27 | 14.82 | 3.45 |
| Ischemic heart disease | 16.74 | 12.99 | 12.53 | 0.46 |
| Tracheal, bronchus, and lung cancer | 8.90 | 6.11 | 6.04 | 0.07 |
| Liver cancer | 3.38 | 5.04 | 4.99 | 0.05 |
| Stomach cancer | 0.40 | 4.85 | 4.78 | 0.07 |
| Alzheimer's disease and other dementias | 4.26 | 3.91 | 3.02 | 0.89 |
| Diabetes mellitus | 5.07 | 3.54 | 1.29 | 2.25 |
| Hypertensive heart disease | 0.75 | 3.33 | 3.15 | 0.19 |
| Esophageal cancer | 0.39 | 2.91 | 2.88 | 0.04 |
| Age-related and other hearing loss | 3.66 | 2.80 | 0.00 | 2.80 |
| Neck pain | 3.92 | 2.65 | 0.00 | 2.65 |
| Cirrhosis and other chronic liver diseases | -0.66 | 2.48 | 2.36 | 0.12 |
| Low back pain | 1.35 | 2.08 | 0.00 | 2.08 |
| Depressive disorders | 2.29 | 2.04 | 0.00 | 2.04 |
| Blindness and vision impairment | 2.36 | 2.00 | 0.00 | 2.00 |
| Chronic kidney disease | 0.66 | 1.92 | 1.44 | 0.49 |
| Colon and rectum cancer | 2.13 | 1.71 | 1.64 | 0.07 |
| Other musculoskeletal disorders | 2.51 | 1.67 | 0.02 | 1.65 |
| Upper digestive system diseases | 0.41 | 1.62 | 0.87 | 0.75 |
| Rheumatic heart disease | -2.18 | 1.32 | 1.28 | 0.04 |
| Tuberculosis | -5.26 | 1.22 | 1.00 | 0.22 |
| Oral disorders | 1.62 | 1.12 | 0.00 | 1.12 |
| Breast cancer | 1.28 | 1.01 | 0.94 | 0.07 |
| Self-harm | -6.64 | 0.92 | 0.90 | 0.02 |
| Falls | 2.03 | 0.92 | 0.38 | 0.54 |
| Headache disorders | 1.76 | 0.82 | 0.00 | 0.82 |
| Osteoarthritis | 1.13 | 0.81 | 0.00 | 0.81 |
| Parkinson's disease | 0.78 | 0.72 | 0.55 | 0.16 |
| Schizophrenia | 1.32 | 0.71 | 0.00 | 0.71 |
| Pancreatic cancer | 1.22 | 0.70 | 0.69 | 0.01 |
| Atrial fibrillation and flutter | 0.72 | 0.56 | 0.29 | 0.27 |
| Cervical cancer | 0.54 | 0.54 | 0.53 | 0.02 |
| Road injuries | -0.54 | 0.50 | -0.16 | 0.66 |
| Other mental disorders | 0.81 | 0.48 | 0.00 | 0.48 |
| Alcohol use disorders | 1.25 | 0.47 | 0.17 | 0.30 |
| Anxiety disorders | 0.86 | 0.42 | 0.00 | 0.42 |
| Nasopharynx cancer | -0.10 | 0.42 | 0.41 | 0.01 |
| Prostate cancer | 0.51 | 0.42 | 0.39 | 0.03 |
| Other malignant neoplasms | 0.43 | 0.41 | 0.41 | 0.00 |
| Gynecological diseases | 0.69 | 0.34 | 0.00 | 0.34 |
| Other cardiovascular and circulatory diseases | 0.33 | 0.33 | 0.21 | 0.13 |
| Urinary diseases and male infertility | -0.04 | 0.32 | 0.10 | 0.22 |
| Asthma | -0.70 | 0.32 | 0.43 | -0.11 |
| Bladder cancer | 0.23 | 0.30 | 0.29 | 0.02 |
| Gallbladder and biliary tract cancer | 0.30 | 0.25 | 0.25 | 0.00 |
| Non-Hodgkin lymphoma | 0.41 | 0.25 | 0.24 | 0.01 |
| Rheumatoid arthritis | 0.34 | 0.24 | 0.08 | 0.16 |
| Gallbladder and biliary diseases | -0.17 | 0.23 | 0.23 | 0.00 |
| Brain and nervous system cancer | 0.15 | 0.23 | 0.23 | 0.00 |
| Larynx cancer | 0.16 | 0.23 | 0.22 | 0.01 |
| Ovarian cancer | 0.43 | 0.22 | 0.21 | 0.01 |
| Uterine cancer | -0.02 | 0.21 | 0.19 | 0.01 |
| Lip and oral cavity cancer | 0.31 | 0.18 | 0.18 | 0.01 |
| Other sense organ diseases | 0.26 | 0.17 | 0.00 | 0.17 |
| Inflammatory bowel disease | 0.16 | 0.15 | 0.04 | 0.11 |
| Pneumoconiosis | 0.00 | 0.15 | 0.13 | 0.02 |
| Bipolar disorder | 0.29 | 0.14 | 0.00 | 0.14 |
| Dietary iron deficiency | -2.58 | 0.14 | 0.00 | 0.14 |
| Aortic aneurysm | 0.14 | 0.14 | 0.14 | 0.00 |
| Other transport injuries | -0.38 | 0.14 | -0.01 | 0.15 |
| Psoriasis | 0.36 | 0.14 | 0.00 | 0.14 |
| Pancreatitis | 0.09 | 0.13 | 0.11 | 0.02 |
| Acute hepatitis | -0.73 | 0.12 | 0.12 | 0.00 |
| Other skin and subcutaneous diseases | 0.31 | 0.12 | 0.00 | 0.12 |
| Non-melanoma skin cancer | 0.18 | 0.12 | 0.12 | 0.00 |
| HIV/AIDS | 1.32 | 0.12 | 0.11 | 0.01 |
| Drug use disorders | -0.30 | 0.12 | 0.18 | -0.07 |
| Fungal skin diseases | 0.14 | 0.12 | 0.00 | 0.12 |
| Non-rheumatic valvular heart disease | 0.07 | 0.11 | 0.10 | 0.02 |
| Multiple myeloma | 0.15 | 0.11 | 0.11 | 0.00 |
| Kidney cancer | 0.22 | 0.11 | 0.10 | 0.00 |
| Exposure to mechanical forces | 0.10 | 0.10 | -0.08 | 0.18 |
| Gout | 0.14 | 0.10 | 0.00 | 0.10 |
| Interstitial lung disease and pulmonary sarcoidosis | 0.17 | 0.10 | 0.05 | 0.05 |
| Other digestive diseases | -0.45 | 0.09 | 0.06 | 0.03 |
| Food-borne trematodiases | 0.15 | 0.09 | 0.00 | 0.09 |
| Peripheral artery disease | 0.09 | 0.08 | 0.01 | 0.07 |
| Environmental heat and cold exposure | -0.40 | 0.08 | 0.01 | 0.07 |
| Cysticercosis | -0.04 | 0.07 | 0.00 | 0.07 |
| Thyroid cancer | 0.08 | 0.07 | 0.06 | 0.01 |
| Acute glomerulonephritis | -0.19 | 0.06 | 0.06 | 0.00 |
| Other pharynx cancer | 0.05 | 0.06 | 0.06 | 0.00 |
| Vascular intestinal disorders | 0.04 | 0.05 | 0.05 | 0.00 |
| Other neoplasms | 0.18 | 0.05 | 0.05 | 0.00 |
| Other chronic respiratory diseases | 0.11 | 0.05 | 0.02 | 0.04 |
| Malignant skin melanoma | 0.05 | 0.05 | 0.05 | 0.00 |
| Other unintentional injuries | 0.10 | 0.04 | -0.01 | 0.06 |
| Hodgkin lymphoma | -0.17 | 0.03 | 0.03 | 0.00 |
| Decubitus ulcer | 0.01 | 0.03 | 0.02 | 0.01 |
| Mesothelioma | 0.04 | 0.03 | 0.03 | 0.00 |
| Pruritus | 0.06 | 0.03 | 0.00 | 0.03 |
| Multiple sclerosis | 0.02 | 0.02 | 0.02 | 0.00 |
| Schistosomiasis | -0.04 | 0.02 | 0.02 | 0.01 |
| Iodine deficiency | 0.01 | 0.02 | 0.00 | 0.02 |
| Cardiomyopathy and myocarditis | 0.12 | 0.02 | 0.01 | 0.01 |
| Trachoma | -0.01 | 0.02 | 0.00 | 0.02 |
| Appendicitis | -0.13 | 0.02 | 0.02 | 0.00 |
| Endocrine, metabolic, blood, and immune disorders | -0.34 | 0.01 | -0.04 | 0.05 |
| Motor neuron disease | 0.04 | 0.01 | 0.01 | 0.00 |
| Alopecia areata | 0.02 | 0.01 | 0.00 | 0.01 |
| Testicular cancer | -0.02 | 0.00 | 0.00 | 0.00 |
| Bacterial skin diseases | -0.10 | 0.00 | 0.00 | 0.00 |
| Executions and police conflict | -0.22 | 0.00 | -0.01 | 0.01 |
| Leprosy | 0.00 | 0.00 | 0.00 | 0.00 |
| Fire, heat, and hot substances | -0.69 | 0.00 | -0.09 | 0.09 |
| Cystic echinococcosis | 0.00 | 0.00 | 0.00 | 0.00 |
| Conflict and terrorism | 0.00 | 0.00 | 0.00 | 0.00 |
| African trypanosomiasis | 0.00 | 0.00 | 0.00 | 0.00 |
| Chagas disease | 0.00 | 0.00 | 0.00 | 0.00 |
| Ebola | 0.00 | 0.00 | 0.00 | 0.00 |
| Guinea worm disease | 0.00 | 0.00 | 0.00 | 0.00 |
| Lymphatic filariasis | 0.00 | 0.00 | 0.00 | 0.00 |
| Onchocerciasis | 0.00 | 0.00 | 0.00 | 0.00 |
| Yellow fever | 0.00 | 0.00 | 0.00 | 0.00 |
| Zika virus | 0.00 | 0.00 | 0.00 | 0.00 |
| Endocarditis | -0.07 | 0.00 | 0.00 | 0.00 |
| Varicella and herpes zoster | -0.12 | 0.00 | -0.02 | 0.02 |
| Animal contact | -0.36 | 0.00 | -0.02 | 0.02 |
| Malaria | -0.01 | 0.00 | 0.00 | 0.00 |
| Dengue | 0.02 | 0.00 | 0.00 | 0.00 |
| Exposure to forces of nature | -0.02 | 0.00 | 0.00 | 0.00 |
| Diphtheria | -0.02 | -0.01 | -0.01 | 0.00 |
| Other intestinal infectious diseases | -0.03 | -0.01 | -0.01 | 0.00 |
| Invasive Non-typhoidal Salmonella (iNTS) | -0.04 | -0.01 | -0.01 | 0.00 |
| Rabies | -0.07 | -0.01 | -0.01 | 0.00 |
| Other neglected tropical diseases | -0.18 | -0.01 | -0.01 | -0.01 |
| Leishmaniasis | -0.14 | -0.02 | -0.02 | 0.00 |
| Inguinal, femoral, and abdominal hernia | 0.09 | -0.02 | 0.01 | -0.03 |
| Idiopathic developmental intellectual disability | -0.01 | -0.03 | 0.00 | -0.03 |
| Other nutritional deficiencies | -0.12 | -0.03 | -0.03 | 0.00 |
| Intestinal nematode infections | -0.77 | -0.03 | -0.01 | -0.02 |
| Other neurological disorders | -0.03 | -0.03 | -0.03 | -0.01 |
| Autism spectrum disorders | 0.10 | -0.05 | 0.00 | -0.05 |
| Otitis media | -0.13 | -0.05 | 0.00 | -0.05 |
| Eating disorders | 0.21 | -0.05 | 0.00 | -0.05 |
| Attention-deficit/hyperactivity disorder | -0.01 | -0.05 | 0.00 | -0.05 |
| Hemoglobinopathies and hemolytic anemias | -1.08 | -0.06 | -0.07 | 0.01 |
| Adverse effects of medical treatment | -0.50 | -0.09 | -0.11 | 0.02 |
| Acne vulgaris | 0.09 | -0.10 | 0.00 | -0.10 |
| Other unspecified infectious diseases | -0.55 | -0.10 | -0.10 | 0.00 |
| Interpersonal violence | -1.53 | -0.10 | -0.27 | 0.16 |
| Maternal disorders | -1.26 | -0.11 | -0.10 | -0.01 |
| Sudden infant death syndrome | -0.26 | -0.11 | -0.11 | 0.00 |
| Poisonings | -0.38 | -0.12 | -0.13 | 0.01 |
| Urticaria | 0.01 | -0.12 | 0.00 | -0.12 |
| Upper respiratory infections | -0.58 | -0.13 | -0.05 | -0.07 |
| Typhoid and paratyphoid | -0.16 | -0.13 | -0.13 | 0.00 |
| Dermatitis | 0.07 | -0.15 | 0.00 | -0.15 |
| Scabies | -0.05 | -0.15 | 0.00 | -0.15 |
| Epilepsy | -0.71 | -0.16 | -0.13 | -0.03 |
| Paralytic ileus and intestinal obstruction | -0.81 | -0.18 | -0.18 | 0.00 |
| Encephalitis | -0.59 | -0.21 | -0.21 | 0.00 |
| Sexually transmitted infections excluding HIV | -0.48 | -0.23 | -0.25 | 0.02 |
| Viral skin diseases | -0.12 | -0.26 | 0.00 | -0.26 |
| Leukemia | -1.57 | -0.30 | -0.30 | 0.00 |
| Tetanus | -1.66 | -0.33 | -0.33 | 0.00 |
| Vitamin A deficiency | -1.00 | -0.38 | 0.00 | -0.38 |
| Conduct disorder | -0.24 | -0.42 | 0.00 | -0.42 |
| Protein-energy malnutrition | -2.42 | -0.52 | -0.51 | -0.01 |
| Meningitis | -2.33 | -0.59 | -0.59 | 0.00 |
| Measles | -2.43 | -0.67 | -0.66 | -0.01 |
| Whooping cough | -2.98 | -0.78 | -0.77 | -0.01 |
| Foreign body | -1.69 | -0.78 | -0.83 | 0.05 |
| Diarrheal diseases | -7.59 | -1.94 | -1.85 | -0.09 |
| Drowning | -9.49 | -2.88 | -2.89 | 0.01 |
| Congenital birth defects | -9.07 | -4.58 | -4.50 | -0.08 |
| Lower respiratory infections | -33.39 | -7.82 | -7.80 | -0.02 |
| Neonatal disorders | -20.61 | -9.67 | -9.01 | -0.66 |

Table S2. Total DALYs and DALYs, YLLs, YLDs associated with population aging between 1990 and 2017 by type of disease for males in China.

| Type of disease | Change in DALYs  （in million) | Estimates associated with population ageing | | |
| --- | --- | --- | --- | --- |
|  |  | DALYs  (in million) | YLLs  (in million) | YLDs  (in million) |
| Stroke | 10.22 | 13.33 | 12.12 | 1.21 |
| Chronic obstructive pulmonary disease | -2.58 | 9.98 | 8.49 | 1.49 |
| Ischemic heart disease | 10.42 | 7.57 | 7.36 | 0.21 |
| Tracheal, bronchus, and lung cancer | 6.30 | 4.31 | 4.26 | 0.05 |
| Liver cancer | 2.96 | 3.65 | 3.61 | 0.04 |
| Stomach cancer | 0.72 | 3.31 | 3.26 | 0.05 |
| Esophageal cancer | 0.61 | 2.07 | 2.05 | 0.02 |
| Diabetes mellitus | 2.94 | 1.78 | 0.61 | 1.18 |
| Hypertensive heart disease | 0.65 | 1.71 | 1.63 | 0.07 |
| Cirrhosis and other chronic liver diseases | -0.29 | 1.70 | 1.63 | 0.07 |
| Alzheimer's disease and other dementias | 1.78 | 1.56 | 1.20 | 0.36 |
| Age-related and other hearing loss | 1.80 | 1.41 | 0.00 | 1.41 |
| Neck pain | 1.56 | 1.06 | 0.00 | 1.06 |
| Colon and rectum cancer | 1.43 | 0.99 | 0.95 | 0.04 |
| Chronic kidney disease | 0.50 | 0.98 | 0.76 | 0.22 |
| Blindness and vision impairment | 1.09 | 0.92 | 0.00 | 0.92 |
| Tuberculosis | -3.06 | 0.90 | 0.75 | 0.15 |
| Upper digestive system diseases | 0.04 | 0.89 | 0.59 | 0.30 |
| Low back pain | 0.53 | 0.78 | 0.00 | 0.78 |
| Other musculoskeletal disorders | 0.90 | 0.77 | 0.01 | 0.76 |
| Depressive disorders | 0.92 | 0.75 | 0.00 | 0.75 |
| Self-harm | -2.23 | 0.56 | 0.55 | 0.01 |
| Falls | 1.19 | 0.53 | 0.24 | 0.29 |
| Rheumatic heart disease | -0.70 | 0.49 | 0.47 | 0.02 |
| Oral disorders | 0.64 | 0.47 | 0.00 | 0.47 |
| Prostate cancer | 0.51 | 0.45 | 0.42 | 0.03 |
| Parkinson's disease | 0.50 | 0.43 | 0.34 | 0.09 |
| Pancreatic cancer | 0.71 | 0.41 | 0.40 | 0.00 |
| Alcohol use disorders | 1.09 | 0.40 | 0.16 | 0.25 |
| Road injuries | -0.35 | 0.38 | -0.01 | 0.39 |
| Schizophrenia | 0.63 | 0.34 | 0.00 | 0.34 |
| Osteoarthritis | 0.41 | 0.31 | 0.00 | 0.31 |
| Nasopharynx cancer | -0.02 | 0.29 | 0.29 | 0.01 |
| Headache disorders | 0.61 | 0.29 | 0.00 | 0.29 |
| Urinary diseases and male infertility | 0.10 | 0.28 | 0.06 | 0.21 |
| Other mental disorders | 0.46 | 0.27 | 0.00 | 0.27 |
| Other malignant neoplasms | 0.28 | 0.27 | 0.25 | 0.02 |
| Atrial fibrillation and flutter | 0.31 | 0.24 | 0.10 | 0.13 |
| Bladder cancer | 0.21 | 0.23 | 0.22 | 0.01 |
| Asthma | -0.41 | 0.22 | 0.28 | -0.06 |
| Larynx cancer | 0.15 | 0.19 | 0.18 | 0.01 |
| Other cardiovascular and circulatory diseases | 0.20 | 0.18 | 0.12 | 0.06 |
| Non-Hodgkin lymphoma | 0.35 | 0.17 | 0.16 | 0.00 |
| Pneumoconiosis | 0.00 | 0.14 | 0.12 | 0.02 |
| Anxiety disorders | 0.31 | 0.14 | 0.00 | 0.14 |
| Brain and nervous system cancer | 0.24 | 0.14 | 0.14 | 0.00 |
| Lip and oral cavity cancer | 0.28 | 0.13 | 0.13 | 0.00 |
| Gallbladder and biliary tract cancer | 0.15 | 0.12 | 0.12 | 0.00 |
| HIV/AIDS | 1.00 | 0.10 | 0.09 | 0.01 |
| Aortic aneurysm | 0.12 | 0.10 | 0.10 | 0.00 |
| Gallbladder and biliary diseases | -0.05 | 0.10 | 0.10 | 0.00 |
| Acute hepatitis | -0.44 | 0.10 | 0.10 | 0.00 |
| Exposure to mechanical forces | 0.06 | 0.09 | -0.03 | 0.12 |
| Drug use disorders | -0.12 | 0.08 | 0.12 | -0.03 |
| Other sense organ diseases | 0.12 | 0.08 | 0.00 | 0.08 |
| Psoriasis | 0.19 | 0.08 | 0.00 | 0.08 |
| Kidney cancer | 0.17 | 0.08 | 0.07 | 0.00 |
| Pancreatitis | 0.07 | 0.07 | 0.06 | 0.01 |
| Bipolar disorder | 0.14 | 0.07 | 0.00 | 0.07 |
| Gout | 0.10 | 0.07 | 0.00 | 0.07 |
| Non-melanoma skin cancer | 0.09 | 0.07 | 0.07 | 0.00 |
| Other transport injuries | -0.24 | 0.07 | 0.00 | 0.07 |
| Rheumatoid arthritis | 0.09 | 0.06 | 0.03 | 0.03 |
| Inflammatory bowel disease | 0.06 | 0.06 | 0.02 | 0.04 |
| Interstitial lung disease and pulmonary sarcoidosis | 0.11 | 0.06 | 0.03 | 0.03 |
| Non-rheumatic valvular heart disease | 0.04 | 0.06 | 0.05 | 0.01 |
| Environmental heat and cold exposure | -0.29 | 0.06 | 0.01 | 0.05 |
| Other skin and subcutaneous diseases | 0.15 | 0.06 | 0.00 | 0.06 |
| Multiple myeloma | 0.10 | 0.06 | 0.06 | 0.00 |
| Fungal skin diseases | 0.06 | 0.05 | 0.00 | 0.05 |
| Food-borne trematodiases | 0.08 | 0.05 | 0.00 | 0.05 |
| Other digestive diseases | -0.24 | 0.05 | 0.04 | 0.01 |
| Other pharynx cancer | 0.05 | 0.05 | 0.05 | 0.00 |
| Peripheral artery disease | 0.05 | 0.04 | 0.01 | 0.03 |
| Other unintentional injuries | 0.16 | 0.04 | 0.00 | 0.04 |
| Acute glomerulonephritis | -0.09 | 0.04 | 0.04 | 0.00 |
| Thyroid cancer | 0.06 | 0.03 | 0.03 | 0.00 |
| Cysticercosis | -0.02 | 0.03 | 0.00 | 0.03 |
| Other chronic respiratory diseases | 0.04 | 0.03 | 0.01 | 0.02 |
| Other neoplasms | 0.13 | 0.03 | 0.03 | 0.00 |
| Vascular intestinal disorders | 0.02 | 0.03 | 0.03 | 0.00 |
| Malignant skin melanoma | 0.02 | 0.03 | 0.02 | 0.00 |
| Cardiomyopathy and myocarditis | 0.13 | 0.02 | 0.02 | 0.01 |
| Breast cancer | 0.07 | 0.02 | 0.02 | 0.00 |
| Hodgkin lymphoma | -0.10 | 0.02 | 0.02 | 0.00 |
| Decubitus ulcer | 0.01 | 0.01 | 0.01 | 0.00 |
| Mesothelioma | 0.03 | 0.01 | 0.01 | 0.00 |
| Iodine deficiency | 0.02 | 0.01 | 0.00 | 0.01 |
| Dietary iron deficiency | -0.97 | 0.01 | 0.00 | 0.01 |
| Multiple sclerosis | 0.01 | 0.01 | 0.01 | 0.00 |
| Schistosomiasis | -0.02 | 0.01 | 0.01 | 0.00 |
| Fire, heat, and hot substances | -0.37 | 0.01 | -0.04 | 0.05 |
| Pruritus | 0.03 | 0.01 | 0.00 | 0.01 |
| Appendicitis | -0.07 | 0.01 | 0.01 | 0.00 |
| Trachoma | 0.00 | 0.01 | 0.00 | 0.01 |
| Inguinal, femoral, and abdominal hernia | 0.09 | 0.01 | 0.01 | 0.00 |
| Testicular cancer | -0.02 | 0.00 | 0.00 | 0.00 |
| Motor neuron disease | 0.02 | 0.00 | 0.00 | 0.00 |
| Bacterial skin diseases | -0.05 | 0.00 | 0.00 | 0.00 |
| Alopecia areata | 0.01 | 0.00 | 0.00 | 0.00 |
| Endocarditis | -0.03 | 0.00 | 0.00 | 0.00 |
| Executions and police conflict | -0.21 | 0.00 | -0.01 | 0.01 |
| Leprosy | 0.00 | 0.00 | 0.00 | 0.00 |
| Animal contact | -0.27 | 0.00 | -0.01 | 0.01 |
| Conflict and terrorism | 0.00 | 0.00 | 0.00 | 0.00 |
| Cystic echinococcosis | 0.00 | 0.00 | 0.00 | 0.00 |
| Guinea worm disease | 0.00 | 0.00 | 0.00 | 0.00 |
| Lymphatic filariasis | 0.00 | 0.00 | 0.00 | 0.00 |
| Onchocerciasis | 0.00 | 0.00 | 0.00 | 0.00 |
| African trypanosomiasis | 0.00 | 0.00 | 0.00 | 0.00 |
| Cervical cancer | 0.00 | 0.00 | 0.00 | 0.00 |
| Chagas disease | 0.00 | 0.00 | 0.00 | 0.00 |
| Ebola | 0.00 | 0.00 | 0.00 | 0.00 |
| Gynecological diseases | 0.00 | 0.00 | 0.00 | 0.00 |
| Maternal disorders | 0.00 | 0.00 | 0.00 | 0.00 |
| Ovarian cancer | 0.00 | 0.00 | 0.00 | 0.00 |
| Uterine cancer | 0.00 | 0.00 | 0.00 | 0.00 |
| Yellow fever | 0.00 | 0.00 | 0.00 | 0.00 |
| Zika virus | 0.00 | 0.00 | 0.00 | 0.00 |
| Malaria | -0.01 | 0.00 | 0.00 | 0.00 |
| Dengue | 0.01 | 0.00 | 0.00 | 0.00 |
| Varicella and herpes zoster | -0.06 | 0.00 | -0.01 | 0.01 |
| Exposure to forces of nature | -0.01 | 0.00 | 0.00 | 0.00 |
| Diphtheria | -0.01 | 0.00 | 0.00 | 0.00 |
| Other intestinal infectious diseases | -0.01 | 0.00 | 0.00 | 0.00 |
| Endocrine, metabolic, blood, and immune disorders | -0.09 | 0.00 | -0.03 | 0.02 |
| Invasive Non-typhoidal Salmonella (iNTS) | -0.02 | -0.01 | -0.01 | 0.00 |
| Rabies | -0.04 | -0.01 | -0.01 | 0.00 |
| Other neglected tropical diseases | -0.09 | -0.01 | 0.00 | -0.01 |
| Leishmaniasis | -0.09 | -0.01 | -0.01 | 0.00 |
| Other nutritional deficiencies | -0.05 | -0.01 | -0.01 | 0.00 |
| Idiopathic developmental intellectual disability | -0.01 | -0.01 | 0.00 | -0.01 |
| Intestinal nematode infections | -0.38 | -0.02 | -0.01 | -0.01 |
| Eating disorders | 0.07 | -0.02 | 0.00 | -0.02 |
| Other neurological disorders | -0.02 | -0.02 | -0.02 | 0.00 |
| Otitis media | -0.07 | -0.03 | 0.00 | -0.03 |
| Autism spectrum disorders | 0.08 | -0.03 | 0.00 | -0.03 |
| Attention-deficit/hyperactivity disorder | -0.01 | -0.04 | 0.00 | -0.04 |
| Interpersonal violence | -1.11 | -0.04 | -0.15 | 0.11 |
| Adverse effects of medical treatment | -0.24 | -0.04 | -0.05 | 0.01 |
| Acne vulgaris | 0.04 | -0.04 | 0.00 | -0.04 |
| Hemoglobinopathies and hemolytic anemias | -0.48 | -0.04 | -0.03 | -0.02 |
| Urticaria | 0.00 | -0.05 | 0.00 | -0.05 |
| Other unspecified infectious diseases | -0.32 | -0.06 | -0.05 | 0.00 |
| Dermatitis | 0.04 | -0.06 | 0.00 | -0.06 |
| Poisonings | -0.26 | -0.06 | -0.06 | 0.00 |
| Upper respiratory infections | -0.30 | -0.06 | -0.03 | -0.04 |
| Typhoid and paratyphoid | -0.09 | -0.07 | -0.07 | 0.00 |
| Sudden infant death syndrome | -0.17 | -0.07 | -0.07 | 0.00 |
| Scabies | -0.03 | -0.08 | 0.00 | -0.08 |
| Epilepsy | -0.40 | -0.09 | -0.07 | -0.02 |
| Paralytic ileus and intestinal obstruction | -0.46 | -0.10 | -0.10 | 0.00 |
| Encephalitis | -0.32 | -0.11 | -0.11 | 0.00 |
| Viral skin diseases | -0.05 | -0.11 | 0.00 | -0.11 |
| Sexually transmitted infections excluding HIV | -0.31 | -0.15 | -0.15 | 0.01 |
| Leukemia | -0.80 | -0.16 | -0.16 | 0.00 |
| Vitamin A deficiency | -0.44 | -0.16 | 0.00 | -0.16 |
| Tetanus | -1.06 | -0.20 | -0.20 | 0.00 |
| Protein-energy malnutrition | -1.01 | -0.21 | -0.20 | -0.01 |
| Conduct disorder | -0.16 | -0.26 | 0.00 | -0.26 |
| Meningitis | -1.36 | -0.33 | -0.33 | 0.00 |
| Measles | -1.27 | -0.34 | -0.33 | 0.00 |
| Whooping cough | -1.41 | -0.35 | -0.35 | 0.00 |
| Foreign body | -0.79 | -0.38 | -0.41 | 0.03 |
| Diarrheal diseases | -3.91 | -0.97 | -0.92 | -0.05 |
| Drowning | -6.28 | -1.87 | -1.87 | 0.01 |
| Congenital birth defects | -5.00 | -2.45 | -2.40 | -0.04 |
| Lower respiratory infections | -16.68 | -3.78 | -3.77 | -0.01 |
| Neonatal disorders | -11.60 | -5.06 | -4.77 | -0.28 |

Table S3. Total DALYs and DALYs, YLLs, YLDs associated with population ageing between 1990 and 2017 by type of disease for females in China.

| Type of disease | Change in DALYs  （in million) | Estimates associated with population ageing | | |
| --- | --- | --- | --- | --- |
|  |  | DALYs  (in million) | YLLs  (in million) | YLDs  (in million) |
| Stroke | 3.91 | 10.33 | 9.03 | 1.29 |
| Chronic obstructive pulmonary disease | -3.94 | 8.40 | 6.45 | 1.95 |
| Ischemic heart disease | 6.33 | 5.42 | 5.17 | 0.25 |
| Alzheimer's disease and other dementias | 2.48 | 2.30 | 1.78 | 0.52 |
| Tracheal, bronchus, and lung cancer | 2.61 | 1.83 | 1.81 | 0.02 |
| Diabetes mellitus | 2.14 | 1.74 | 0.68 | 1.06 |
| Hypertensive heart disease | 0.10 | 1.62 | 1.51 | 0.11 |
| Neck pain | 2.36 | 1.60 | 0.00 | 1.60 |
| Stomach cancer | -0.32 | 1.57 | 1.55 | 0.02 |
| Age-related and other hearing loss | 1.85 | 1.39 | 0.00 | 1.39 |
| Liver cancer | 0.42 | 1.33 | 1.31 | 0.01 |
| Depressive disorders | 1.37 | 1.30 | 0.00 | 1.30 |
| Low back pain | 0.82 | 1.30 | 0.00 | 1.30 |
| Blindness and vision impairment | 1.26 | 1.07 | 0.00 | 1.07 |
| Breast cancer | 1.21 | 1.01 | 0.94 | 0.07 |
| Chronic kidney disease | 0.16 | 0.95 | 0.69 | 0.26 |
| Other musculoskeletal disorders | 1.62 | 0.91 | 0.01 | 0.89 |
| Esophageal cancer | -0.22 | 0.85 | 0.83 | 0.01 |
| Rheumatic heart disease | -1.48 | 0.84 | 0.81 | 0.03 |
| Cirrhosis and other chronic liver diseases | -0.37 | 0.74 | 0.70 | 0.04 |
| Upper digestive system diseases | 0.38 | 0.73 | 0.29 | 0.45 |
| Colon and rectum cancer | 0.70 | 0.73 | 0.70 | 0.03 |
| Oral disorders | 0.98 | 0.65 | 0.00 | 0.65 |
| Cervical cancer | 0.54 | 0.55 | 0.54 | 0.02 |
| Headache disorders | 1.14 | 0.54 | 0.00 | 0.54 |
| Osteoarthritis | 0.72 | 0.50 | 0.00 | 0.50 |
| Falls | 0.83 | 0.38 | 0.13 | 0.25 |
| Gynecological diseases | 0.69 | 0.37 | 0.00 | 0.37 |
| Self-harm | -4.41 | 0.37 | 0.36 | 0.01 |
| Schizophrenia | 0.68 | 0.36 | 0.00 | 0.36 |
| Tuberculosis | -2.19 | 0.32 | 0.25 | 0.07 |
| Atrial fibrillation and flutter | 0.41 | 0.32 | 0.18 | 0.14 |
| Parkinson's disease | 0.29 | 0.30 | 0.23 | 0.07 |
| Pancreatic cancer | 0.51 | 0.29 | 0.29 | 0.00 |
| Anxiety disorders | 0.55 | 0.27 | 0.00 | 0.27 |
| Ovarian cancer | 0.43 | 0.22 | 0.22 | 0.01 |
| Uterine cancer | -0.02 | 0.21 | 0.20 | 0.01 |
| Other mental disorders | 0.35 | 0.21 | 0.00 | 0.21 |
| Rheumatoid arthritis | 0.26 | 0.18 | 0.05 | 0.13 |
| Other cardiovascular and circulatory diseases | 0.14 | 0.15 | 0.09 | 0.06 |
| Other malignant neoplasms | 0.14 | 0.14 | 0.16 | -0.02 |
| Dietary iron deficiency | -1.61 | 0.14 | 0.00 | 0.14 |
| Gallbladder and biliary tract cancer | 0.15 | 0.13 | 0.13 | 0.00 |
| Gallbladder and biliary diseases | -0.12 | 0.13 | 0.13 | 0.00 |
| Road injuries | -0.18 | 0.12 | -0.15 | 0.27 |
| Nasopharynx cancer | -0.09 | 0.12 | 0.12 | 0.00 |
| Asthma | -0.29 | 0.11 | 0.16 | -0.05 |
| Brain and nervous system cancer | -0.09 | 0.10 | 0.09 | 0.00 |
| Other sense organ diseases | 0.14 | 0.09 | 0.00 | 0.09 |
| Inflammatory bowel disease | 0.10 | 0.09 | 0.02 | 0.07 |
| Non-Hodgkin lymphoma | 0.06 | 0.08 | 0.08 | 0.00 |
| Bladder cancer | 0.02 | 0.08 | 0.08 | 0.00 |
| Bipolar disorder | 0.15 | 0.07 | 0.00 | 0.07 |
| Other transport injuries | -0.14 | 0.07 | -0.02 | 0.09 |
| Other skin and subcutaneous diseases | 0.16 | 0.06 | 0.00 | 0.06 |
| Fungal skin diseases | 0.08 | 0.06 | 0.00 | 0.06 |
| Psoriasis | 0.17 | 0.06 | 0.00 | 0.06 |
| Pancreatitis | 0.02 | 0.06 | 0.04 | 0.01 |
| Non-rheumatic valvular heart disease | 0.03 | 0.05 | 0.04 | 0.01 |
| Non-melanoma skin cancer | 0.09 | 0.05 | 0.05 | 0.00 |
| Alcohol use disorders | 0.17 | 0.05 | 0.01 | 0.04 |
| Lip and oral cavity cancer | 0.03 | 0.05 | 0.05 | 0.00 |
| Urinary diseases and male infertility | -0.13 | 0.05 | 0.04 | 0.01 |
| Multiple myeloma | 0.06 | 0.05 | 0.05 | 0.00 |
| Other digestive diseases | -0.21 | 0.04 | 0.02 | 0.02 |
| Peripheral artery disease | 0.04 | 0.04 | 0.01 | 0.04 |
| Larynx cancer | 0.01 | 0.04 | 0.04 | 0.00 |
| Aortic aneurysm | 0.02 | 0.04 | 0.04 | 0.00 |
| Interstitial lung disease and pulmonary sarcoidosis | 0.07 | 0.04 | 0.02 | 0.02 |
| Thyroid cancer | 0.01 | 0.03 | 0.03 | 0.00 |
| Cysticercosis | -0.02 | 0.03 | 0.00 | 0.03 |
| Kidney cancer | 0.05 | 0.03 | 0.03 | 0.00 |
| Food-borne trematodiases | 0.07 | 0.03 | 0.00 | 0.03 |
| Gout | 0.04 | 0.03 | 0.00 | 0.03 |
| Vascular intestinal disorders | 0.02 | 0.03 | 0.03 | 0.00 |
| Other neoplasms | 0.05 | 0.03 | 0.02 | 0.00 |
| Acute glomerulonephritis | -0.10 | 0.02 | 0.02 | 0.00 |
| Drug use disorders | -0.18 | 0.02 | 0.06 | -0.04 |
| Malignant skin melanoma | 0.03 | 0.02 | 0.02 | 0.00 |
| Other chronic respiratory diseases | 0.07 | 0.02 | 0.00 | 0.02 |
| Acute hepatitis | -0.29 | 0.02 | 0.02 | 0.00 |
| Environmental heat and cold exposure | -0.11 | 0.02 | 0.00 | 0.02 |
| Pruritus | 0.03 | 0.01 | 0.00 | 0.01 |
| HIV/AIDS | 0.32 | 0.01 | 0.01 | 0.00 |
| Endocrine, metabolic, blood, and immune disorders | -0.25 | 0.01 | -0.02 | 0.03 |
| Decubitus ulcer | 0.01 | 0.01 | 0.01 | 0.00 |
| Multiple sclerosis | 0.01 | 0.01 | 0.01 | 0.00 |
| Schistosomiasis | -0.02 | 0.01 | 0.01 | 0.00 |
| Trachoma | -0.01 | 0.01 | 0.00 | 0.01 |
| Other pharynx cancer | 0.00 | 0.01 | 0.01 | 0.00 |
| Mesothelioma | 0.01 | 0.01 | 0.01 | 0.00 |
| Hodgkin lymphoma | -0.06 | 0.01 | 0.01 | 0.00 |
| Iodine deficiency | -0.01 | 0.01 | 0.00 | 0.01 |
| Pneumoconiosis | -0.01 | 0.01 | 0.01 | 0.00 |
| Appendicitis | -0.06 | 0.01 | 0.01 | 0.00 |
| Exposure to mechanical forces | 0.04 | 0.01 | -0.05 | 0.06 |
| Other unintentional injuries | -0.06 | 0.01 | -0.02 | 0.02 |
| Alopecia areata | 0.01 | 0.00 | 0.00 | 0.00 |
| Motor neuron disease | 0.01 | 0.00 | 0.00 | 0.00 |
| Varicella and herpes zoster | -0.05 | 0.00 | -0.01 | 0.01 |
| Leprosy | 0.00 | 0.00 | 0.00 | 0.00 |
| Cystic echinococcosis | 0.00 | 0.00 | 0.00 | 0.00 |
| Guinea worm disease | 0.00 | 0.00 | 0.00 | 0.00 |
| Lymphatic filariasis | 0.00 | 0.00 | 0.00 | 0.00 |
| Onchocerciasis | 0.00 | 0.00 | 0.00 | 0.00 |
| African trypanosomiasis | 0.00 | 0.00 | 0.00 | 0.00 |
| Chagas disease | 0.00 | 0.00 | 0.00 | 0.00 |
| Ebola | 0.00 | 0.00 | 0.00 | 0.00 |
| Prostate cancer | 0.00 | 0.00 | 0.00 | 0.00 |
| Testicular cancer | 0.00 | 0.00 | 0.00 | 0.00 |
| Yellow fever | 0.00 | 0.00 | 0.00 | 0.00 |
| Zika virus | 0.00 | 0.00 | 0.00 | 0.00 |
| Conflict and terrorism | 0.00 | 0.00 | 0.00 | 0.00 |
| Bacterial skin diseases | -0.05 | 0.00 | 0.00 | 0.00 |
| Animal contact | -0.09 | 0.00 | -0.01 | 0.01 |
| Executions and police conflict | 0.00 | 0.00 | 0.00 | 0.00 |
| Malaria | -0.01 | 0.00 | 0.00 | 0.00 |
| Dengue | 0.01 | 0.00 | 0.00 | 0.00 |
| Exposure to forces of nature | -0.01 | 0.00 | 0.00 | 0.00 |
| Endocarditis | -0.04 | 0.00 | 0.00 | 0.00 |
| Diphtheria | -0.01 | 0.00 | 0.00 | 0.00 |
| Other neglected tropical diseases | -0.10 | 0.00 | 0.00 | 0.00 |
| Cardiomyopathy and myocarditis | -0.01 | 0.00 | -0.01 | 0.01 |
| Other intestinal infectious diseases | -0.02 | 0.00 | 0.00 | 0.00 |
| Rabies | -0.03 | -0.01 | -0.01 | 0.00 |
| Invasive Non-typhoidal Salmonella (iNTS) | -0.02 | -0.01 | -0.01 | 0.00 |
| Leishmaniasis | -0.04 | -0.01 | -0.01 | 0.00 |
| Other neurological disorders | -0.02 | -0.01 | 0.00 | 0.00 |
| Autism spectrum disorders | 0.02 | -0.01 | 0.00 | -0.01 |
| Hemoglobinopathies and hemolytic anemias | -0.60 | -0.01 | -0.04 | 0.03 |
| Fire, heat, and hot substances | -0.32 | -0.01 | -0.06 | 0.05 |
| Idiopathic developmental intellectual disability | 0.00 | -0.01 | 0.00 | -0.01 |
| Attention-deficit/hyperactivity disorder | 0.00 | -0.01 | 0.00 | -0.01 |
| Other nutritional deficiencies | -0.07 | -0.02 | -0.02 | 0.00 |
| Intestinal nematode infections | -0.39 | -0.02 | -0.01 | -0.01 |
| Inguinal, femoral, and abdominal hernia | 0.01 | -0.02 | 0.01 | -0.02 |
| Otitis media | -0.06 | -0.02 | 0.00 | -0.02 |
| Eating disorders | 0.14 | -0.03 | 0.00 | -0.03 |
| Sudden infant death syndrome | -0.09 | -0.04 | -0.04 | 0.00 |
| Other unspecified infectious diseases | -0.24 | -0.04 | -0.04 | 0.00 |
| Adverse effects of medical treatment | -0.26 | -0.05 | -0.06 | 0.01 |
| Acne vulgaris | 0.05 | -0.06 | 0.00 | -0.06 |
| Typhoid and paratyphoid | -0.07 | -0.06 | -0.06 | 0.00 |
| Poisonings | -0.12 | -0.06 | -0.06 | 0.00 |
| Upper respiratory infections | -0.28 | -0.07 | -0.03 | -0.04 |
| Interpersonal violence | -0.42 | -0.07 | -0.12 | 0.05 |
| Urticaria | 0.01 | -0.07 | 0.00 | -0.07 |
| Scabies | -0.02 | -0.07 | 0.00 | -0.07 |
| Epilepsy | -0.30 | -0.08 | -0.06 | -0.02 |
| Sexually transmitted infections excluding HIV | -0.17 | -0.08 | -0.09 | 0.02 |
| Paralytic ileus and intestinal obstruction | -0.35 | -0.08 | -0.08 | 0.00 |
| Dermatitis | 0.03 | -0.09 | 0.00 | -0.09 |
| Encephalitis | -0.27 | -0.10 | -0.10 | 0.00 |
| Maternal disorders | -1.26 | -0.11 | -0.10 | -0.01 |
| Tetanus | -0.60 | -0.13 | -0.13 | 0.00 |
| Leukemia | -0.77 | -0.13 | -0.13 | 0.00 |
| Conduct disorder | -0.08 | -0.14 | 0.00 | -0.14 |
| Viral skin diseases | -0.07 | -0.16 | 0.00 | -0.16 |
| Vitamin A deficiency | -0.56 | -0.23 | 0.00 | -0.23 |
| Meningitis | -0.97 | -0.26 | -0.26 | 0.00 |
| Protein-energy malnutrition | -1.41 | -0.32 | -0.31 | -0.01 |
| Measles | -1.15 | -0.33 | -0.33 | 0.00 |
| Foreign body | -0.90 | -0.40 | -0.42 | 0.02 |
| Whooping cough | -1.57 | -0.43 | -0.43 | 0.00 |
| Drowning | -3.20 | -0.93 | -0.94 | 0.01 |
| Diarrheal diseases | -3.68 | -0.97 | -0.93 | -0.04 |
| Congenital birth defects | -4.07 | -2.11 | -2.08 | -0.04 |
| Lower respiratory infections | -16.71 | -4.03 | -4.02 | -0.01 |
| Neonatal disorders | -9.01 | -4.60 | -4.21 | -0.39 |

Table S4. DALYs associated with changes in DALY rate attributed to risk factors versus population ageing between 1990 and 2017 for both sexes in China.

| Type of disease | DALYs associated with population ageing | DALYs associated with changes in DALY rate attributed to risk factors | DALYs associated with changes in unattributed DALY rate |
| --- | --- | --- | --- |
| **Completely counteracted** |  |  |  |
| Chronic obstructive pulmonary disease | 18.27 | -22.97 | -6.31 |
| Hypertensive heart disease | 3.33 | -3.38 | 0.00 |
| Chronic kidney disease | 1.92 | -2.02 | 0.00 |
| Tuberculosis | 1.22 | -3.04 | -4.23 |
| Self-harm | 0.92 | -1.36 | -7.51 |
| Road injuries | 0.50 | -2.51 | -0.76 |
| Asthma | 0.32 | -0.37 | -0.99 |
| Pneumoconiosis | 0.15 | -0.20 | 0.00 |
| Dietary iron deficiency | 0.14 | -3.16 | 0.00 |
| Other transport injuries | 0.14 | -0.45 | -0.28 |
| Drug use disorders | 0.12 | -1.37 | 0.00 |
| Exposure to mechanical forces | 0.10 | -0.40 | 0.05 |
| Other unintentional injuries | 0.04 | -0.18 | 0.06 |
| Fire, heat, and hot substances | 0.00 | -0.31 | -0.56 |
| **Partially counteracted** |  |  |  |
| Stroke | 23.58 | -13.06 | -2.71 |
| Liver cancer | 5.04 | -0.06 | -3.17 |
| Stomach cancer | 4.85 | -3.77 | -2.03 |
| Esophageal cancer | 2.91 | -2.13 | -1.15 |
| Age-related and other hearing loss | 2.80 | -0.07 | -0.10 |
| Cirrhosis and other chronic liver diseases | 2.48 | -1.06 | -2.94 |
| Low back pain | 2.08 | -1.35 | -0.47 |
| Depressive disorders | 2.04 | -0.11 | -0.85 |
| Blindness and vision impairment | 2.00 | -0.07 | -0.28 |
| Upper digestive system diseases | 1.62 | -0.43 | -1.44 |
| Rheumatic heart disease | 1.32 | -0.69 | -3.35 |
| Falls | 0.92 | -0.31 | 0.63 |
| Parkinson's disease | 0.72 | -0.02 | -0.08 |
| Cervical cancer | 0.54 | -0.18 | 0.00 |
| Nasopharynx cancer | 0.42 | -0.31 | -0.36 |
| Bladder cancer | 0.30 | -0.04 | -0.11 |
| Gallbladder and biliary diseases | 0.23 | -0.06 | -0.41 |
| Larynx cancer | 0.23 | -0.08 | -0.06 |
| Aortic aneurysm | 0.14 | -0.01 | -0.04 |
| Acute hepatitis | 0.12 | 0.00 | -0.99 |
| Peripheral artery disease | 0.08 | 0.00 | -0.01 |
| Environmental heat and cold exposure | 0.08 | -0.03 | -0.55 |
| Other pharynx cancer | 0.06 | -0.01 | -0.02 |
| Multiple sclerosis | 0.02 | 0.00 | -0.01 |
| **No counteracting effect** |  |  |  |
| Ischemic heart disease | 12.99 | 0.42 | -0.13 |
| Tracheal, bronchus, and lung cancer | 6.11 | 0.93 | 0.16 |
| Alzheimer's disease and other dementias | 3.91 | 0.15 | -0.58 |
| Diabetes mellitus | 3.54 | 0.33 | 0.00 |
| Colon and rectum cancer | 1.71 | 0.09 | -0.18 |
| Breast cancer | 1.01 | 0.14 | -0.19 |
| Osteoarthritis | 0.81 | 0.14 | -0.04 |
| Pancreatic cancer | 0.70 | 0.16 | 0.16 |
| Atrial fibrillation and flutter | 0.56 | 0.07 | -0.04 |
| Alcohol use disorders | 0.47 | 0.44 | 0.00 |
| Anxiety disorders | 0.42 | 0.02 | -0.21 |
| Prostate cancer | 0.42 | 0.00 | -0.01 |
| Other cardiovascular and circulatory diseases | 0.33 | 0.04 | -0.19 |
| Gallbladder and biliary tract cancer | 0.25 | 0.03 | -0.05 |
| Non-Hodgkin lymphoma | 0.25 | 0.02 | -0.01 |
| Rheumatoid arthritis | 0.24 | 0.00 | 0.02 |
| Ovarian cancer | 0.22 | 0.02 | 0.12 |
| Uterine cancer | 0.21 | 0.00 | -0.29 |
| Lip and oral cavity cancer | 0.18 | 0.09 | -0.03 |
| Pancreatitis | 0.13 | 0.02 | -0.10 |
| HIV/AIDS | 0.12 | 0.86 | 0.21 |
| Non-rheumatic valvular heart disease | 0.11 | 0.00 | -0.08 |
| Multiple myeloma | 0.11 | 0.01 | 0.00 |
| Kidney cancer | 0.11 | 0.05 | 0.01 |
| Gout | 0.10 | 0.02 | -0.01 |
| Thyroid cancer | 0.07 | 0.01 | -0.02 |
| Mesothelioma | 0.03 | 0.01 | 0.00 |
| Cardiomyopathy and myocarditis | 0.02 | 0.06 | -0.08 |

Notes：Only 66 diseases with increased DALYs associated with population ageing for both sexes were presented.

Table S5. DALYs associated with changes in DALY rate attributed to risk factors versus population ageing between 1990 and 2017 for males in China

| Type of disease | DALYs associated with population ageing | DALYs associated with changes in DALY rate attributed to risk factors | DALYs associated with changes in unattributed DALY rate |
| --- | --- | --- | --- |
| **Completely counteracted** |  |  |  |
| Chronic obstructive pulmonary disease | 9.98 | -14.73 | -2.50 |
| Tuberculosis | 0.90 | -4.43 | -1.75 |
| Self-harm | 0.56 | -3.38 | -2.47 |
| Road injuries | 0.38 | -2.25 | -0.52 |
| Asthma | 0.22 | -0.81 | -0.51 |
| Pneumoconiosis | 0.14 | -0.18 | 0.00 |
| Exposure to mechanical forces | 0.09 | -0.28 | 0.05 |
| Drug use disorders | 0.08 | -0.82 | 0.00 |
| Other transport injuries | 0.07 | -0.42 | -0.17 |
| Other unintentional injuries | 0.04 | 0.00 | 0.11 |
| Dietary iron deficiency | 0.01 | -1.12 | 0.00 |
| Fire, heat, and hot substances | 0.01 | -0.48 | -0.30 |
| Animal contact | 0.00 | -0.31 | -0.19 |
| **Partially counteracted** |  |  |  |
| Stroke | 13.33 | -5.18 | -1.26 |
| Stomach cancer | 3.31 | -2.23 | -1.20 |
| Esophageal cancer | 2.07 | -1.53 | -0.45 |
| Hypertensive heart disease | 1.71 | -1.44 | 0.00 |
| Cirrhosis and other chronic liver diseases | 1.70 | -0.76 | -1.81 |
| Age-related and other hearing loss | 1.41 | -0.05 | -0.05 |
| Chronic kidney disease | 0.98 | -0.85 | 0.00 |
| Upper digestive system diseases | 0.89 | -0.41 | -0.79 |
| Low back pain | 0.78 | -0.60 | -0.10 |
| Falls | 0.53 | -0.27 | 0.45 |
| Rheumatic heart disease | 0.49 | -0.23 | -1.15 |
| Parkinson's disease | 0.43 | -0.01 | -0.01 |
| Nasopharynx cancer | 0.29 | -0.26 | -0.15 |
| Bladder cancer | 0.23 | -0.02 | -0.05 |
| Larynx cancer | 0.19 | -0.06 | -0.03 |
| Aortic aneurysm | 0.10 | 0.00 | -0.01 |
| Gallbladder and biliary diseases | 0.10 | -0.03 | -0.15 |
| Acute hepatitis | 0.10 | 0.00 | -0.62 |
| Environmental heat and cold exposure | 0.06 | -0.03 | -0.39 |
| Other pharynx cancer | 0.05 | -0.01 | -0.01 |
| Multiple sclerosis | 0.01 | 0.00 | 0.00 |
| **No counteracting effect** |  |  |  |
| Ischemic heart disease | 7.57 | 1.00 | -0.09 |
| Tracheal, bronchus, and lung cancer | 4.31 | 1.02 | -0.15 |
| Liver cancer | 3.65 | 0.04 | -1.84 |
| Diabetes mellitus | 1.78 | 0.55 | 0.00 |
| Alzheimer's disease and other dementias | 1.56 | 0.05 | -0.12 |
| Colon and rectum cancer | 0.99 | 0.21 | -0.05 |
| Blindness and vision impairment | 0.92 | 0.01 | -0.16 |
| Depressive disorders | 0.75 | 0.00 | -0.26 |
| Prostate cancer | 0.45 | 0.00 | -0.04 |
| Pancreatic cancer | 0.41 | 0.12 | 0.07 |
| Alcohol use disorders | 0.40 | 0.42 | 0.00 |
| Osteoarthritis | 0.31 | 0.05 | -0.02 |
| Atrial fibrillation and flutter | 0.24 | 0.04 | -0.02 |
| Other cardiovascular and circulatory diseases | 0.18 | 0.03 | -0.10 |
| Non-Hodgkin lymphoma | 0.17 | 0.02 | 0.08 |
| Anxiety disorders | 0.14 | 0.01 | -0.07 |
| Lip and oral cavity cancer | 0.13 | 0.11 | 0.00 |
| Gallbladder and biliary tract cancer | 0.12 | 0.01 | -0.01 |
| HIV/AIDS | 0.10 | 0.65 | 0.15 |
| Kidney cancer | 0.08 | 0.04 | 0.03 |
| Pancreatitis | 0.07 | 0.02 | -0.05 |
| Gout | 0.07 | 0.02 | -0.01 |
| Rheumatoid arthritis | 0.06 | 0.00 | 0.00 |
| Non-rheumatic valvular heart disease | 0.06 | 0.00 | -0.04 |
| Multiple myeloma | 0.06 | 0.00 | 0.01 |
| Peripheral artery disease | 0.04 | 0.00 | 0.00 |
| Thyroid cancer | 0.03 | 0.00 | 0.02 |
| Cardiomyopathy and myocarditis | 0.02 | 0.06 | -0.02 |
| Breast cancer | 0.02 | 0.01 | 0.03 |
| Mesothelioma | 0.01 | 0.01 | 0.00 |
| Endocarditis | 0.00 | 0.01 | -0.06 |

Notes：Only 65 diseases with increased DALYs associated with population ageing for males were presented.

Table S6. DALYs associated with changes in DALY rate attributed to risk factors versus population ageing between 1990 and 2017 for females in China

| Type of disease | DALYs associated with population ageing | DALYs associated with changes in DALY rate attributed to risk factors | DALYs associated with changes in unattributed DALY rate |
| --- | --- | --- | --- |
| **Completely counteracted** |  |  |  |
| Chronic obstructive pulmonary disease | 8.40 | -10.89 | -3.76 |
| Hypertensive heart disease | 1.62 | -1.94 | 0.00 |
| Chronic kidney disease | 0.95 | -1.18 | 0.00 |
| Self-harm | 0.37 | -0.43 | -5.06 |
| Tuberculosis | 0.32 | -0.35 | -2.49 |
| Dietary iron deficiency | 0.14 | -2.06 | 0.00 |
| Road injuries | 0.12 | -0.73 | -0.23 |
| Other transport injuries | 0.07 | -0.20 | -0.11 |
| Drug use disorders | 0.02 | -0.52 | 0.00 |
| Pneumoconiosis | 0.01 | -0.02 | 0.00 |
| Exposure to mechanical forces | 0.01 | -0.07 | 0.01 |
| Other unintentional injuries | 0.01 | -0.06 | -0.05 |
| **Partially counteracted** |  |  |  |
| Stroke | 10.33 | -7.90 | -1.46 |
| Ischemic heart disease | 5.42 | -0.54 | -0.04 |
| Tracheal, bronchus, and lung cancer | 1.83 | -0.08 | 0.31 |
| Diabetes mellitus | 1.74 | -0.20 | 0.00 |
| Stomach cancer | 1.57 | -1.53 | -0.84 |
| Age-related and other hearing loss | 1.39 | -0.02 | -0.05 |
| Liver cancer | 1.33 | -0.06 | -1.27 |
| Depressive disorders | 1.30 | -0.12 | -0.61 |
| Low back pain | 1.30 | -0.76 | -0.37 |
| Blindness and vision impairment | 1.07 | -0.08 | -0.12 |
| Esophageal cancer | 0.85 | -0.59 | -0.71 |
| Rheumatic heart disease | 0.84 | -0.46 | -2.21 |
| Cirrhosis and other chronic liver diseases | 0.74 | -0.28 | -1.09 |
| Upper digestive system diseases | 0.73 | -0.02 | -0.65 |
| Colon and rectum cancer | 0.73 | -0.12 | -0.14 |
| Cervical cancer | 0.55 | -0.20 | 0.00 |
| Falls | 0.38 | -0.01 | 0.18 |
| Rheumatoid arthritis | 0.18 | 0.00 | 0.02 |
| Gallbladder and biliary diseases | 0.13 | -0.03 | -0.26 |
| Nasopharynx cancer | 0.12 | -0.05 | -0.21 |
| Asthma | 0.11 | -0.07 | -0.49 |
| Bladder cancer | 0.08 | -0.02 | -0.07 |
| Non-rheumatic valvular heart disease | 0.05 | 0.00 | -0.04 |
| Lip and oral cavity cancer | 0.05 | -0.01 | -0.03 |
| Peripheral artery disease | 0.04 | 0.00 | 0.00 |
| Larynx cancer | 0.04 | -0.02 | -0.03 |
| Aortic aneurysm | 0.04 | -0.01 | -0.02 |
| Acute hepatitis | 0.02 | 0.00 | -0.36 |
| Environmental heat and cold exposure | 0.02 | 0.00 | -0.16 |
| Multiple sclerosis | 0.01 | 0.00 | -0.01 |
| Other pharynx cancer | 0.01 | 0.00 | -0.01 |
| Mesothelioma | 0.01 | -0.01 | 0.00 |
| **No counteracting effect** |  |  |  |
| Alzheimer's disease and other dementias | 2.30 | 0.07 | -0.40 |
| Breast cancer | 1.01 | 0.12 | -0.25 |
| Osteoarthritis | 0.50 | 0.09 | -0.02 |
| Atrial fibrillation and flutter | 0.32 | 0.04 | -0.02 |
| Parkinson's disease | 0.30 | 0.00 | -0.09 |
| Pancreatic cancer | 0.29 | 0.04 | 0.09 |
| Anxiety disorders | 0.27 | 0.01 | -0.15 |
| Ovarian cancer | 0.22 | 0.02 | 0.11 |
| Uterine cancer | 0.21 | 0.00 | -0.30 |
| Other cardiovascular and circulatory diseases | 0.15 | 0.01 | -0.10 |
| Gallbladder and biliary tract cancer | 0.13 | 0.02 | -0.04 |
| Non-Hodgkin lymphoma | 0.08 | 0.01 | -0.09 |
| Pancreatitis | 0.06 | 0.00 | -0.06 |
| Alcohol use disorders | 0.05 | 0.05 | 0.00 |
| Multiple myeloma | 0.05 | 0.00 | -0.02 |
| Thyroid cancer | 0.03 | 0.00 | -0.03 |
| Kidney cancer | 0.03 | 0.01 | -0.01 |
| Gout | 0.03 | 0.01 | 0.00 |
| HIV/AIDS | 0.01 | 0.21 | 0.06 |

Notes：Only 63 diseases with increased DALYs associated with population ageing for both sexes were presented.

**5. GATHER Checklist**

**
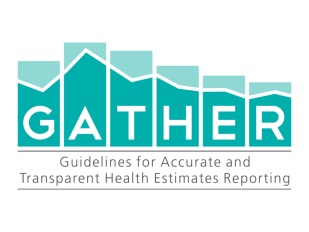
Checklist of information that should be included in new reports of global health estimates**

| Item # | Checklist item | Reported on page # |
| --- | --- | --- |
| Objectives and funding | | |
| 1 | Define the indicator(s), populations (including age, sex, and geographic entities), and time period(s) for which estimates were made. | Methods-paragraph 1 |
| 2 | List the funding sources for the work. | No funding |
| Data Inputs | | |
| *For all data inputs from multiple sources that are synthesized as part of the study:* | | |
| 3 | Describe how the data were identified and how the data were accessed. | Methods-paragraph 1 |
| 4 | Specify the inclusion and exclusion criteria. Identify all ad-hoc exclusions. | No data were excluded (Methods-paragraph 1) |
| 5 | Provide information on all included data sources and their main characteristics. For each data source used, report reference information or contact name/institution, population represented, data collection method, year(s) of data collection, sex and age range, diagnostic criteria or measurement method, and sample size, as relevant. | Methods-paragraph 1 Results-paragraph 1 |
| 6 | Identify and describe any categories of input data that have potentially important biases (e.g., based on characteristics listed in item 5). | No such data (Methods-paragraph 1) |
| *For data inputs that contribute to the analysis but were not synthesized as part of the study:* | | |
| 7 | Describe and give sources for any other data inputs. | All data were derived from GBD 2017 (Methods-paragraph 1) |
| *For all data inputs:* | | |
| 8 | Provide all data inputs in a file format from which data can be efficiently extracted (e.g., a spreadsheet rather than a PDF), including all relevant meta-data listed in item 5. For any data inputs that cannot be shared because of ethical or legal reasons, such as third-party ownership, provide a contact name or the name of the institution that retains the right to the data. | Available from: <http://ghdx.healthdata.org/gbd-results-tool>  and  <https://population.un.org/wpp/Download/Standard/Population/> |
| Data analysis | | |
| 9 | Provide a conceptual overview of the data analysis method. A diagram may be helpful. | Methods-paragraph 3 and appendix 1 |
| 10 | Provide a detailed description of all steps of the analysis, including mathematical formulae. This description should cover, as relevant, data cleaning, data pre-processing, data adjustments and weighting of data sources, and mathematical or statistical model(s). | Methods-paragraph 4 and 5 |
| 11 | Describe how candidate models were evaluated and how the final model(s) were selected. | Only one method was used in this study (Methods-paragraph 3) |
| 12 | Provide the results of an evaluation of model performance, if done, as well as the results of any relevant sensitivity analysis. | The robustness of the method was evaluated in other research (appendix 1) |
| 13 | Describe methods for calculating uncertainty of the estimates. State which sources of uncertainty were, and were not, accounted for in the uncertainty analysis. | Discussion-paragraph 5 |
| 14 | State how analytic or statistical source code used to generate estimates can be accessed. | No specific software was needed for the estimation. |
| Results and Discussion | | |
| 15 | Provide published estimates in a file format from which data can be efficiently extracted. | Appendix (Table.1-1, 1-2, 1-3, 2-1, 2-2, 2-3) |
| 16 | Report a quantitative measure of the uncertainty of the estimates (e.g. uncertainty intervals). | Discussion-paragraph 5 |
| 17 | Interpret results in light of existing evidence. If updating a previous set of estimates, describe the reasons for changes in estimates. | Discussion-paragraph 2, 3, 4 |
| 18 | Discuss limitations of the estimates. Include a discussion of any modelling assumptions or data limitations that affect interpretation of the estimates. | Discussion-paragraph 5 |

*This checklist should be used in conjunction with the GATHER statement and Explanation and Elaboration document, found on gather-statement.org*

**Supplementary References**

1. Cheng XJ, Tan LH, Gao YY, Yang Y, Schwebel DC, Hu GQ. A new method to attribute differences in total deaths between groups to population size, age structure and age-specific mortality rate. PLoS One. 2019;14(5):e0216613.

2. Cheng X, Yang Y, Schwebel DC, et al. Population ageing and mortality during 1990-2017: A global decomposition analysis. PLoS Med. 2020;17(6):e1003138.
